# Design rules for reciprocal coupling in chemically fueled assembly†

**DOI:** 10.1039/d3sc02062b

**Published:** 2023-08-22

**Authors:** Xiaoyao Chen, Brigitte A. K. Kriebisch, Alexander M. Bergmann, Job Boekhoven

**Affiliations:** a Department of Chemistry, School of Natural Sciences, Technical University of Munich Lichtenbergstrasse 4 85748 Garching bei München Germany job.boekhoven@tum.de

## Abstract

Biology regulates the function and assembly of proteins through non-equilibrium reaction cycles. Reciprocally, the assembly of proteins can influence the reaction rates of these cycles. Such reciprocal coupling between assembly and reaction cycle is a prerequisite for behavior like dynamic instabilities, treadmilling, pattern formation, and oscillations between morphologies. While assemblies regulated by chemical reaction cycles gained traction, the concept of reciprocal coupling is under-explored. In this work, we provide two molecular design strategies to tweak the degree of reciprocal coupling between the assembly and reaction cycle. The strategies involve spacing the chemically active site away from the assembly or burying it into the assembly. We envision that design strategies facilitate the creation of reciprocally coupled and, by extension, dynamic supramolecular materials in the future.

## Abstract 2

Chemical reaction cycles can regulate molecular self-assembly. *Vice versa*, self-assembly can regulate reaction rates. Here, we show design rules to tune such reciprocal coupling in dissipative self-assembly.

## Graphical abstract

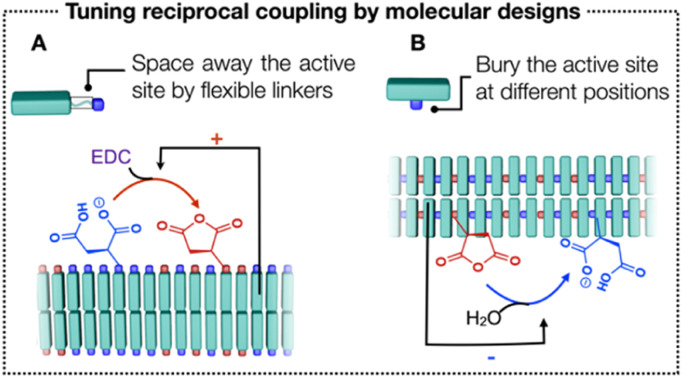

## Introduction

**Scheme 1:**
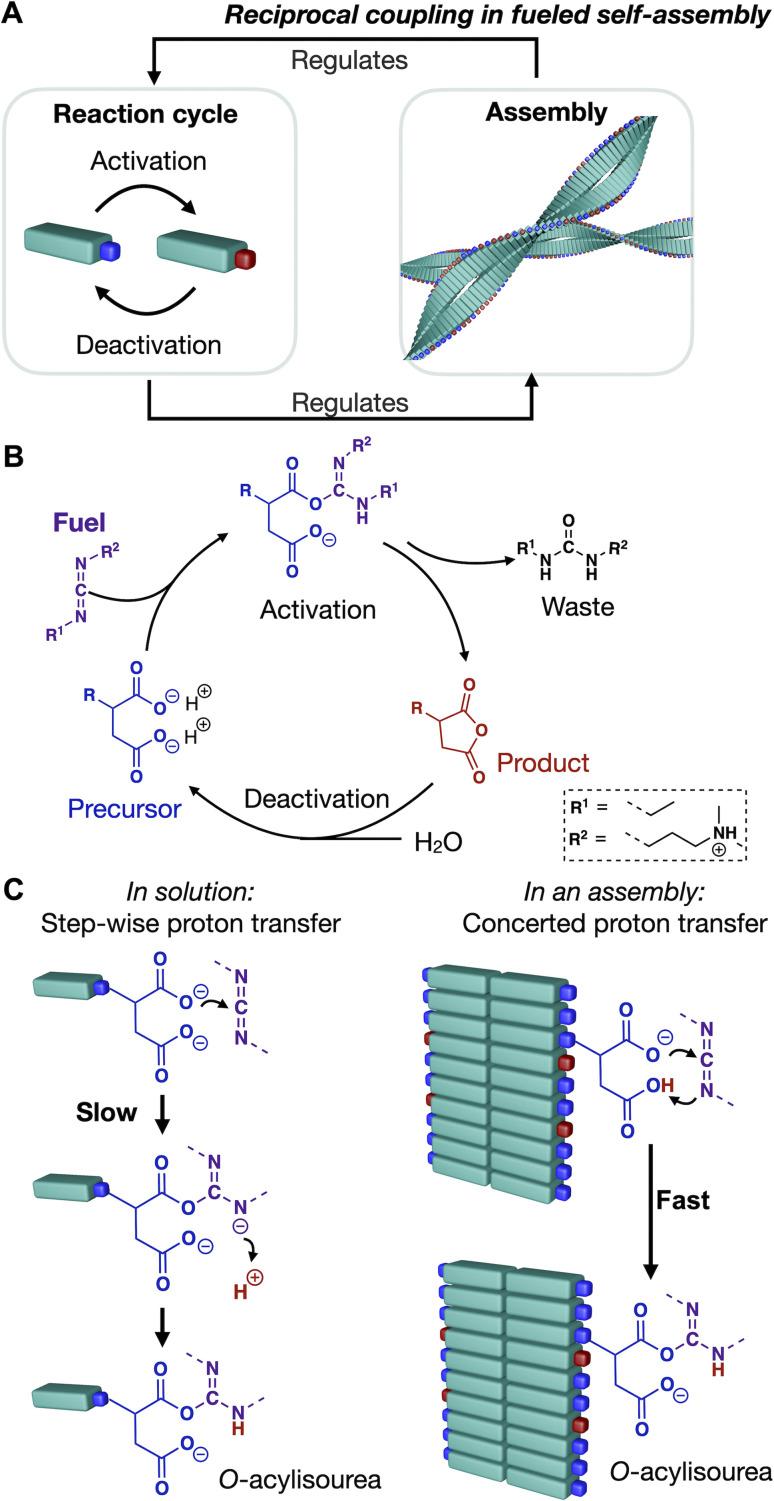
A) Simplified scheme of reciprocal coupling in chemically fueled assemblies. The assembly is regulated by the reaction cycle and *vice versa*. (B) Chemically fueled reaction cycle. (C) Protonation of precursor controls activation reaction pathway: stepwise *vs.* concerted proton transfer.

Biological supramolecular structures like the nucleolus, the transmembrane ATPases, and the cytoskeleton are tightly regulated using non-equilibrium chemical reaction cycles, giving them unique properties like the capacity to heal or form patterns.^1–3^ These reaction cycles are catalytic and convert molecules with high chemical potential into their lower chemical potential counterparts, like the enzymatic conversion of ATP to ADP. Upon doing so, the enzyme is temporarily activated for a function like assembling. Prototypical examples are the ATP-driven formation of actin filaments or ATP-fueled ATPase pumps. *Vice versa*, the resulting assemblies also regulate the activation and deactivation of their building blocks.^4–6^ Thus, the reaction cycle and assembly are reciprocally coupled (Scheme 1A). Again, actin assembly is a great example—ATP activates monomeric, globular actin (G-actin) for assembly into filaments.^4^ Upon assembly, actin changes its conformation (F-actin), resulting in an increased ATP hydrolysis rate.^5^ ATP hydrolysis triggers disassembly. Thus, the reaction cycle activates and deactivates molecules for assembly. *Vice versa*, the assembly affects the kinetics of the reaction cycle by catalyzing hydrolysis—the assembly process and its reaction cycle are reciprocally coupled. In an extreme case of reciprocal coupling, a building block's activation can only occur in the non-assembled state, and its deactivation can only occur in the assembled state.^7^ In such a scenario, building blocks must assemble and disassemble to proceed through a reaction cycle. Such reciprocal coupling is a prerequisite for driven self-assembly and behavior like dynamic instabilities, treadmilling, pattern formation, and oscillations between morphologies. Even though reciprocal coupling is ubiquitous in biology, synthetic assemblies with similar dynamic behavior are underexplored. To create synthetic assemblies with similar dynamic behavior, we must understand mechanisms by which assembly and reaction cycle reciprocally affect each other. Moreover, we need design rules by which we can control the nature of feedback and the degree of reciprocal coupling interaction.

So far, several fuel-driven chemical reaction cycles have been developed—those that are driven by the oxidation of reducing agents and *vice versa*,^8–10^ the hydration of high-energy reagents like carbodiimides,^11–13^ the hydrolysis of methylating agents,^14^ or biomolecules like adenosine triphosphate (ATP).^15,16^ These chemical reaction cycles activate and deactivate molecules for assembly into, *e.g.*, micelles,^9,17,18^ vesicles,^19^ droplets,^12,20^ colloids,^8,11^ nano- or microparticle clusters,^21,22^ and fibers.^11,14,16^ Moreover, molecular assemblies that catalyze chemical reactions are well known and include micelles that catalyze Diels–Alder reactions,^23^ membrane-less organelles catalyze aldol reactions,^24^ catalytic fibers and nanotubes catalyze aldol reactions,^25^ ester hydrolysis,^26,27^ Diels–Alder reactions.^28^ However, systems where the two concepts are combined, *i.e.*, a reaction cycle that regulates assembly and the assembly that regulates the reaction cycle, are underexplored.^10,11,17,29–31^ Design rules are needed to control the nature of feedback and the degree of reciprocal coupling.

We recently found a chemically fueled peptide that forms fibers that display reciprocal coupling between assembly and cycle (Scheme 1).^31^ In the chemical reaction cycle, an anionic dicarboxylate peptide (precursor) converts into a non-charged anhydride (product) (Scheme 1B) by reacting with a carbodiimide. The hydrophobization, upon converting to an anhydride, induces self-assembly into fibers. Simultaneously, hydrolysis of the anhydride reinstates the negative charges on the precursor, which induces disassembly.^11^ Reciprocally, we demonstrated that the assembly into fibers accelerates both the EDC-consuming activation of the peptide and the deactivation of the building block through hydrolysis (Scheme 1C).^31^ The underlying mechanism has to do with the organization of the active sites of the peptide, which affects their apparent p*K*_a_ and thereby changes the reaction pathway. Peptides that do not assemble have a relatively low apparent p*K*_a_ and, thus, a low degree of protonation. Organizing the peptides into fibers increases the degree of protonation of the active sites, affecting whether the rate-determining step of the activation reaction proceeds *via* the slow stepwise or the fast concerted proton transfer pathway (Scheme 1C). Besides, the strength of hydrogen bonding also affects the deactivation rate. We hypothesized that the hydrogen bonding of a neighboring amide and the anhydride carbonyl's oxygen increases the carbonyl carbon's electrophilicity. Thereby, the anhydride hydrolysis is accelerated.

**Fig. 1:**
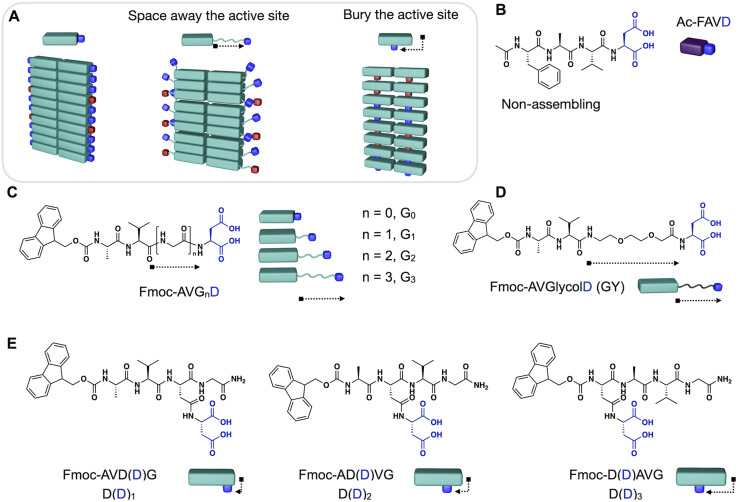
Molecular design strategies to control reciprocal coupling and the nature of feedback. (A) Schematic representation of the design strategies. Either the active site is spaced away or buried in the stack compared to the original design (left). (B–E) Molecular design of precursor to regulate reciprocal coupling in chemically fueled assemblies. (B) The non-assembling compound as a reference. Spacing the aspartic acid away from the peptide core Fmoc–AV with (C) Gly-spacers and (D) an ethylene glycol segment. (E) Bury the aspartic acid in the peptide core by changing its position in the peptide sequence.

In this work, we demonstrate design strategies by which the degree of reciprocal coupling and its nature can be tuned (Fig. 1A). In one peptide, Ac–FAVD, the active site is attached to a non-assembling peptide (Fig. 1B). In one peptide design, Fmoc–AVG_*n*_D, the active site is attached to a peptide that stacks into fibers. We space the chemical active site further from the peptide stack with a glycine spacer and an ethylene glycol spacer (Fig. 1C and D). Finally, we attach the active site to the self-assembling peptide but bury it inside the peptide stack and place it at a different position in the peptide sequence (Fig. 1E). We measure the reciprocal coupling between assembly and reaction kinetics for each design strategy.

## Results and discussion

### The degree of reciprocal coupling as a function of spacer length

**Fig. 2:**
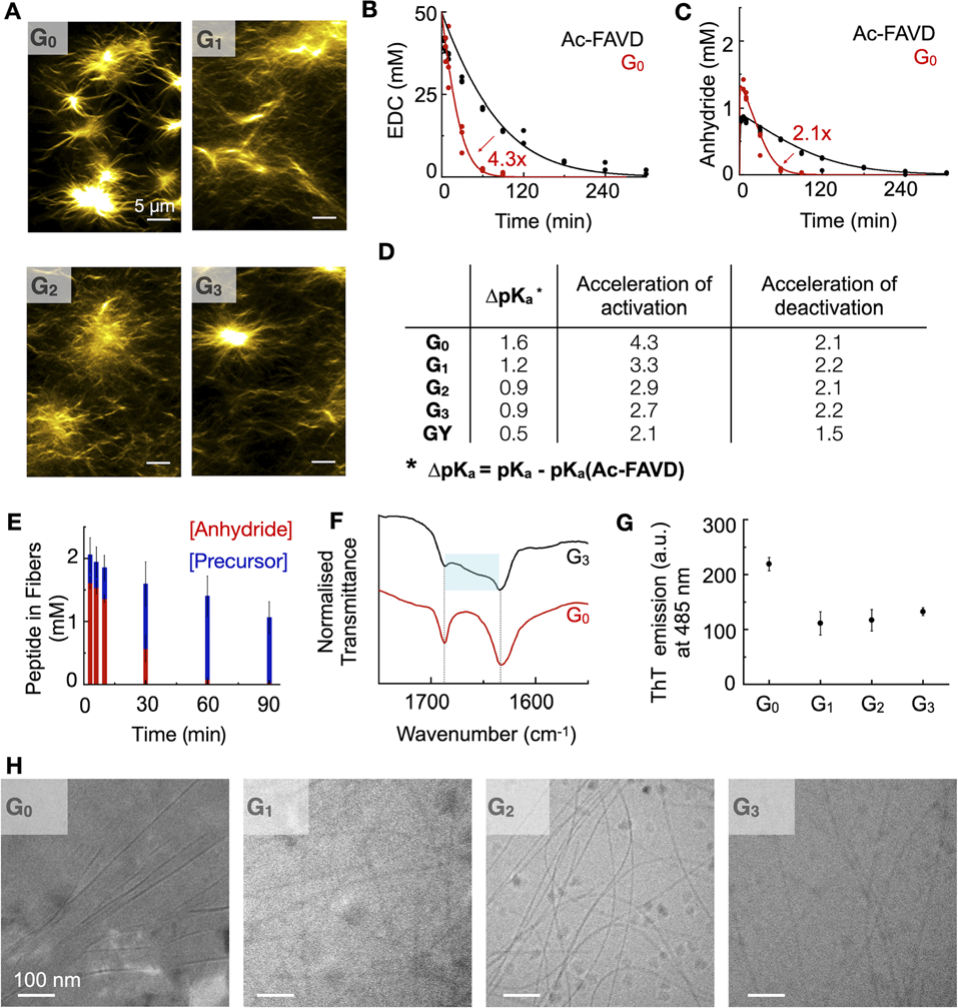
The self-assembly and kinetic behavior of G_*n*_ and GY-peptide compared to Ac–FAVD. (A) Confocal micrographs of 2.5 mM G_*n*_ 10 minutes after fueling with 50 mM EDC. HPLC (markers) and kinetic model data (lines) as a function of time of (B) the EDC consumption for G_0_ and Ac–FAVD, (C) the anhydride concentration of G_0_ and Ac–FAVD after fueling with EDC, *n* = 3. (D) The influence of Gly's number and an ethylene glycol spacer (GY) on (1) the shift of apparent p*K*_a_ of G_*n*_/GY to Ac–FAVD (2) the activation and deactivation reaction rate (*k*_1_, *k*_4_) of G_*n*_/GY compared to Ac–FAVD. (E) The components of the chemically fueled fibers as a function of time determined by ^1^H-NMR spectroscopy. Experiments were done in duplicate. (F) FT-IR spectra of G_0_ and G_3_ fueled with EDC at 3 min. Fmoc–OCNH band at 1687/1686 cm^−1^ and amide I band at 1633/1634 cm^−1^ (typical β-sheet). The blue box highlights the broad shoulder between 1640–1670 cm^−1^. (G) Maximal ThT emission (a.u.) at 485 nm after excitation at 440 nm when 2.5 mM G_*n*_ is fueled with 10 mM EDC, *n* = 3. (H) Cryo-TEM micrographs of 2.5 mM G_*n*_ fueled with 25 mM EDC at 2 min. Scale bar 100 nm.

The internal ordering or rigidity of the chemically fueled fibers results from stacking the hydrophobic Fmoc-group and β-sheet hydrogen bonds between the alanine and valine. To regulate the rigidity's effect on the apparent p*K*_a_ of the carboxylates, which are the active site, we spaced it away from the peptide domain responsible for the fiber's rigidity with a glycine linker. We designed the peptides Fmoc–AVG_*n*_D–OH (G_*n*_) with *n* = 0, 1, 2, 3, *i.e.*, G_0_, G_1_, G_2_, G_3_ (Fig. 1C). We chose glycine (G) as a flexible spacer because it lacks a side group, having the greatest freedom of rotation of the N–C_α_ (phi, *φ*) and C_α_–C′ (psi, *ψ*) and, thus, the lowest propensity to form β-sheets.^32,33^ We dissolved the peptides G_*n*_ at 2.5 mM in an aqueous buffer of 200 mM MES at pH 6. The addition of 50 mM 1-ethyl-3-(3-dimethylaminopropyl)carbodiimide (EDC) initiated the reaction cycle and led to the formation of a dense network of bundled fibers for all peptides (Fig. 2A). We determined the critical aggregation concentration of the peptides by a Nile red fluorescence assay. We used Nile red's increased emission intensity as a proxy for fiber formation. We added increasing amounts of peptide to a solution of 50 mM EDC and measured the fluorescence intensity (Fig. S1A–D†). The peptide needed to get the first evidence of assemblies was 0.5 mM. We then used the kinetic model (*vide infra*) to calculate the amount of anhydride around 0.2–0.3 mM when 0.5 mM precursor is used. Thus, we conclude that the anhydride's critical aggregation concentration (CAC) of the G_*n*_ peptides is below 0.3 mM. We studied the kinetics of the reaction cycle for G_*n*_ by applying fuel and using a benzylamine quench^34^ to stop the reaction cycle at predetermined time points. Benzylamine reacts with the anhydride (the activated peptide) to form the stable benzylamide, and the increase in pH stops the activation reaction. We monitored the concentration of fuel and benzylamide as a measure of the anhydride by high-performance liquid chromatography (HPLC) (Fig. 2B, C, S2A–C, G–I and S3–S7†). The activated peptide was temporarily sustained and decayed as the system ran out of fuel.

To quantify the nature of feedback and the degree of reciprocal coupling between the fibers and the reaction cycle, we used the non-assembling peptide Ac–FAVD–OH (Ac–FAVD, Ac = acetyl, F = phenylalanine) as a reference (Fig. S8A†). We observed that all G_*n*_ consumed EDC faster than Ac–FAVD (Fig. 2B, S2A–C and S3–S7†). Moreover, the anhydride of all G_*n*_ peptides was hydrolyzed faster than Ac–FAVD (Fig. 2C, S2G–I and S3–S7†). A kinetic model was used to fit the data and estimate all rate constants, confirming the acceleration (ESI – Kinetic model†).^11–13,35–37^ The peptide without spacer G_0_ showed the largest increase in the second-order rate constant of activation relative to Ac–FAVD, *i.e.*, a factor of 4.3 (Fig. 2D and Table S3†). In contrast, the acceleration of the activation decreased to 3.3/2.9/2.7 for G_1_, G_2_, G_3_ compared to Ac–FAVD. Moreover, the rate constant of anhydride hydrolysis remained more or less constant with varying Gly spacer lengths and ranged between 2.1–2.2-fold acceleration compared to the non-assembling Ac–FAVD (Fig. 2D and Table S3†).

For the assembly to accelerate the activation, two conditions must be fulfilled.^31^ First, the assembly is a co-assembly of activated and deactivated peptides, *i.e.*, precursors and anhydrides. Second, the apparent p*K*_a_ of the deactivated peptide is shifted in the assembly compared to in solution. Indeed, we found by ^1^H-NMR spectroscopy that the deactivated peptide co-assembled into the fibers in all cases of G_*n*_ (Fig. 2E, S9A–C and S10†). Over the entire 90 minute chemical reaction cycle, most of the peptide was in the self-assembled state. Even though all the anhydride hydrolyzed back to its precursor after 90 minutes, the precursor stayed trapped inside the assembled fibers, indicating that the disassembly rate for all precursors is very slow. Because most of the peptide is in the assembly, we expect the co-assembled peptide to be reactivated inside the fiber. As the co-assembled peptide is exposed to a different microenvironment than in solution, we expect a change in the apparent p*K*_a_ of the active site and, thus, its reactivity.^38,39^

We determined the apparent p*K*_a_ by titration. We found that for the non-assembling Ac–FAVD, the apparent p*K*_a_ values were 4.2 and 2.2, which is in line with reference values for aspartic acid (Fig. S11A†).^40^ In contrast, for all G_*n*_, only a single apparent p*K*_a_ value was found with a shift to higher values, *i.e.*, 5.8 for G_0_, 5.6 for G_1_, 5.1 for G_2_, and 5.1 for G_3_ (Fig. S11B–E†).

Our combined findings conclude that the deactivated peptide is co-assembled with the activated peptide, and the change of its microenvironment compared to the non-assembled peptide changes the apparent p*K*_a_ of the active site. However, the further active site is spaced away from the fiber the smaller the change in apparent p*K*_a_. As the activation reaction is rate limited by a proton transfer, shifting the apparent p*K*_a_ to a higher value increased the activation rate. Yet, the effect decreases with increasing glycine spacer. We hypothesize that more than two Gly-spacers do not lead to more conformational flexibility of the aspartic acid headgroup.

We studied the influence of the Gly spacers on the β-sheet interactions of the fibers by Fourier-Transform Infrared spectroscopy (FT-IR). With FT-IR measurements, the β-sheet interactions are normally characterized in a range of 1625–1640 cm^−1^.^41^ These fibers showed two predominant peaks: the amide I band at 1630 cm^−1^ (carbonyl stretch vibration responsible for the β-sheet interactions) and the Fmoc–OCNH band at 1690 cm^−1^ (Fig. 2F, S12A and B†). The amide I band is sensitive to hydrogen bonding in the assembly, and it broadens towards higher wavenumbers with increasing glycine spacer length. This broadening indicates that the peptide assemblies are less ordered, *i.e.*, the β-sheet interactions between peptides are decreased.^41^ Moreover, we used a ThT assay to measure the assembly's ability to bind ThT, which is a measure of the flatness and, thus, the rigidity of the β-sheets of the assemblies.^42,43^ The maximum emission intensity of ThT decreased by introducing Gly-spacers, indicating a decreased degree of β-sheet flatness and rigidity for G_*n*_ (*n* = 1, 2, 3) assemblies (Fig. 2G, S13A–D and S14A–D†). Cryogenic-Transmission Electron Microscopy (Cryo-TEM) studies showed that G_0_ assembled into broad tapes with a width of 52 ± 11 nm whereas G_*n*_ (*n* = 1, 2, 3) assembled into thin fibers with a width of 7 ± 2 nm (Fig. 2H). The planar tape structure of G_0_ indicates that the active aspartic acid headgroup is conformationally more constrained than in the thin fiber assemblies of G_*n*_ (*n* = 1, 2, 3).

To further improve the flexibility of the spacer unit, we used an ethylene glycol spacer. We designed Fmoc–AV–ethylene glycol-D–OH, *i.e.*, GY (Fig. 1D). We added 50 mM EDC to 2.5 mM GY in MES buffer at pH 6 to initiate the reaction cycle, which led to the formation of fibers (Fig. S8B†). We determined the CAC to be around 0.2 mM (Fig. S1E†). The kinetic experiments showed that the activation was only 2.1-fold accelerated compared to Ac–FAVD. The anhydride hydrolysis rate constant was slightly by a 1.5-fold (Fig. 2D, Table S3, Fig. S2D, J, S4C, S7C and D†). Like G_*n*_, ^1^H-NMR spectroscopy showed that the deactivated peptide co-assembled into the GY fibers (Fig. S9D and S15†). In the fiber microenvironment, the apparent p*K*_a_ shifts to 4.7 (Fig. S11F†). Thus, compared to G_*n*,_ the p*K*_a_-shift is lower, resulting in a lower acceleration of activation and deactivation. The FT-IR-spectra of GY showed next to the characteristic amide I band at 1630 cm^−1^ also a broad shoulder between 1640–1670 cm^−1^ (Fig. S12C†). This peak shape points to a random coil (1640–1650 cm^−1^) next to elements of β-sheet structures (1625–1640 cm^−1^), meaning that parts of the peptide assemblies are even less ordered than the G_*n*_ peptides.^41^

We conclude that introducing Gly-spacers as well as an ethylene glycol-spacer between the peptide core Fmoc–AV and the active site introduces conformational flexibility of the active aspartic acid headgroup and thus decreases the shift in the apparent p*K*_a_ value of the carboxylates. This enables us to tune the acceleration of the activation reaction compared to G_0_. The higher the flexibility of the Gly *vs.* ethylene glycol, the greater the effect on the conformational flexibility of the active aspartic acid headgroup. The effect decreases and flattens if we introduce more than one Gly-spacer.

### Tuning the reciprocal coupling by burying the active site

**Fig. 3:**
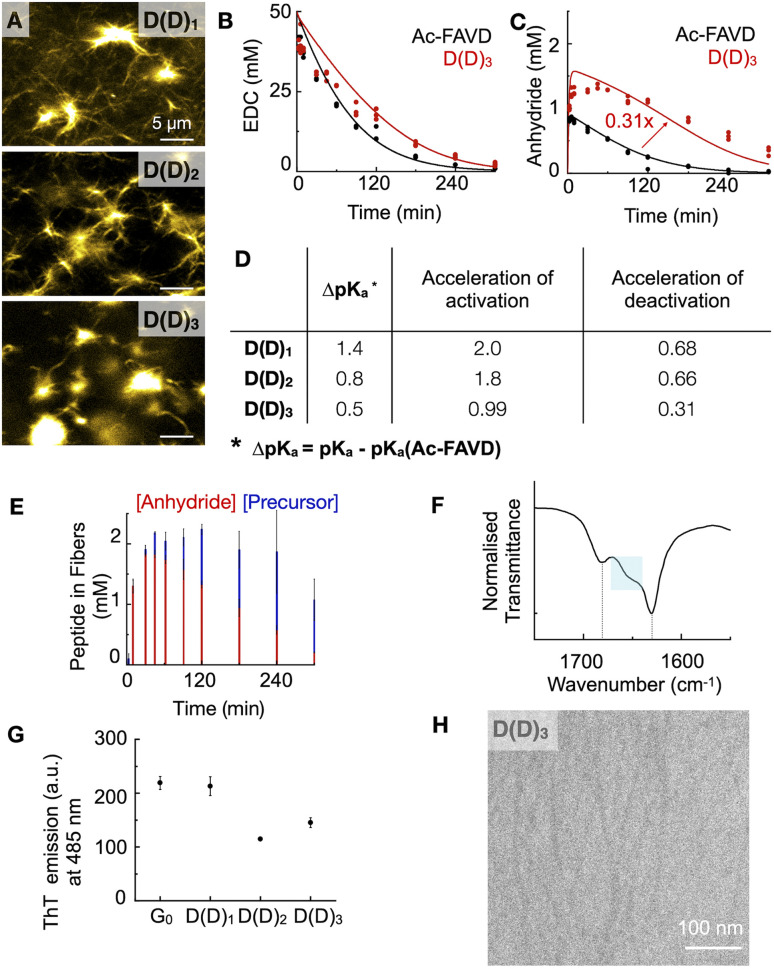
The self-assembly and kinetic behaviour of D(D)_*n*_ compared to Ac–FAVD. (A) Confocal micrographs of D(D)_*n*_ 10 minutes after fueling with EDC. HPLC (markers) and kinetic model data (lines) as a function of time of (B) the EDC consumption for D(D)_3_ and Ac–FAVD and (C) the anhydride concentration of Ac–FAVD and D(D)_3_ after fueling with EDC *n* = 3. (D) The influence of the branched linker on (1) the shift of apparent p*K*_a_ of D(D)_*n*_ to Ac–FAVD; (2) the activation and deactivation reaction rate (*k*_1_, *k*_4_) of D(D)_*n*_ compared to Ac–FAVD. (E) The components of the chemically fueled fibers as a function of time determined by ^1^H-NMR spectroscopy. Experiments were done in duplicate. (F) FT-IR spectra of D(D)_3_ fueled with EDC at 3 min. Fmoc–OCNH band at 1681 cm^−1^ and amide I band at 1630 cm^−1^ (typical β-sheet). The blue box highlights the broad shoulder between 1640–1670 cm^−1^. (G) Maximal ThT emission (a.u.) at 485 nm after excitation at 440 nm when 2.5 mM G_0_/D(D)_*n*_ is fueled with 10 mM EDC, *n* = 3. (H) Cryo-TEM micrographs of 2.5 mM D(D)_3_ fueled with 25 mM EDC at 2 min. Scale bar 100 nm.

We designed the peptides Fmoc–AVD(D)G–NH_2_ (D(D)_1_), Fmoc–AD(D)VG–NH_2_ (D(D)_2_), and Fmoc–D(D)AVG–NH_2_ (D(D)_3_) in which the active site, *i.e.*, the aspartic acid, is placed at different positions in the Fmoc–AVG peptide sequence *via* an aspartic acid linker (Fig. 1E). Thus, the active site is branched away from the peptide chain and buried deeper into the fiber's core with increasing number. We hypothesize that the buried and branched dicarboxylate group disrupts the strong π–π interactions of the Fmoc-group and the β-sheet hydrogen-bonding interactions of the AV segment, thereby influencing the degree of reciprocal coupling. We dissolved 2.5 mM D(D)_*n*_ in MES buffer at pH 6 and added 50 mM EDC to initiate the reaction cycle, which led to the formation of fibers (Fig. 3A). We found the CAC of these peptides to be below 0.3 mM (Fig. S1F–H†).

We analyzed the evolution of the D(D)_*n*_ reaction cycle by HPLC and compared it to the non-assembling Ac–FAVD (Fig. 3B, C, S2E, F, K, L and S16–S18†). The data from our kinetic model showed that the activation rate constant was 2-fold to 1.8-fold accelerated for D(D)_1_ and D(D)_2_ but not accelerated for D(D)_3_ compared to Ac–FAVD (Fig. 3B, D, S2E, F and S16–S18†). ^1^H-NMR spectroscopy combined with HPLC showed that over the entire reaction cycle, the D(D)_*n*_ fibers were a co-assembly of activated and deactivated peptides (Fig. 3E, S9E, F and S19†). As for the G_*n*_ and GY peptides, most of the peptide is trapped inside the assembly, indicating a slow disassembly rate. Thus, we expect the co-assembled D(D)_*n*_ peptide to be reactivated inside the fiber as well. In the fiber microenvironment, the apparent p*K*_a_ changes, and thus the reactivity of the D(D)_*n*_ peptides changes. The apparent p*K*_a_ we measured for D(D)_*n*_ in its assembled state is and 5.6 for D(D)_1_, 5.0 for D(D)_2_ and 4.7 for D(D)_3_ (Fig. S11G–I†). Thus, compared to the non-assembling Ac–FAVD, the apparent p*K*_a_ shifts 0.5–1.4 units higher (Fig. 3D). The further the branched active aspartic acid is spaced away from the Fmoc–fiber core, the less buried the active site is and the higher the apparent p*K*_a_ shift, and thus the greater acceleration of the activation reaction. Indeed, the acceleration of the activation reaction for D(D)_*n*_ decreased from 2.0 to 0.99 from D(D)_1_ to D(D)_3_. In stark contrast to the G_*n*_ peptide series and the GY peptide, we found that the rate constant for activated D(D)_*n*_ deactivation around 30% slower for D(D)_1_ and D(D)_2_ and 70% slower for D(D)_3_, respectively than for Ac–FAVD (Fig. 3C, D, S2K, L and S16–S18†). We hypothesize that the hydrophobic fiber core excludes water. Thus, the buried anhydride, especially in D(D)_3_, is protected from hydrolysis. In D(D)_1_ and D(D)_2_ fibers, the active site is less buried, and thus the anhydride is less protected from hydrolysis. This is in line with confocal microscopy that shows a dense fiber network for D(D)_1_ and D(D)_2_ while for D(D)_3_ we observe more nucleation sites from which fibers grow (Fig. 3A).

In the FTIR-spectra, the D(D)_3_ amide I band peaked at 1633 cm^−1^ and showed a broad shoulder between 1640–1670 cm^−1^ (Fig. 3F). This peak shape points to the presence of β-sheet structures (1625–1640 cm^−1^) next to elements of a random coil (1640–1650 cm^−1^), meaning that parts of the peptide assemblies are less ordered.^41^ Going from D(D)_1_ to D(D)_3,_ the broad shoulder increases, indicating less-ordered assemblies (Fig. 3F, S12D and E†). The maximum emission intensity of ThT for D(D)_1_ is almost equal to G_0_ as the aspartic acid branch does not disrupt the Fmoc-π–π interactions and the hydrogen-bonding interactions of alanine and valine. However, for D(D)_*n*_ (*n* = 2, 3) assemblies, the signal was lower than G_0_. In D(D)_2_ the aspartic acid branch is placed between alanine and valine and we hypothesize it disrupts the hydrogen bonding. In D(D)_3_, the aspartic acid branch disrupts the β-sheet hydrogen-bonding interactions of the AV-segment and the Fmoc-π–π interactions (Fig. 3G, S13A, E–G, S14A and E–G†). Cryo-TEM shows that D(D)_3_ assembled into thin fibers with a width of 6 ± 1 nm (Fig. 3H). The combined dataset of FT-IR, ThT, and Cryo-TEM reveals weaker intermolecular interaction of D(D)_*n*_ compared to G_0_ (FT-IR), yielding less rigid assemblies. Moreover, the more the branched active aspartic acid is placed in the fiber core, D(D)_1_*vs.* D(D)_3_, the less rigid are the assemblies. These findings corroborate our hypothesis that the buried dicarboxylate disrupts the strong π–π interactions of the Fmoc-group and the β-sheet hydrogen-bonding interactions of the peptide sequence AVG.

### Molecular design tunes the nature and degree of reciprocal coupling

**Fig. 4:**
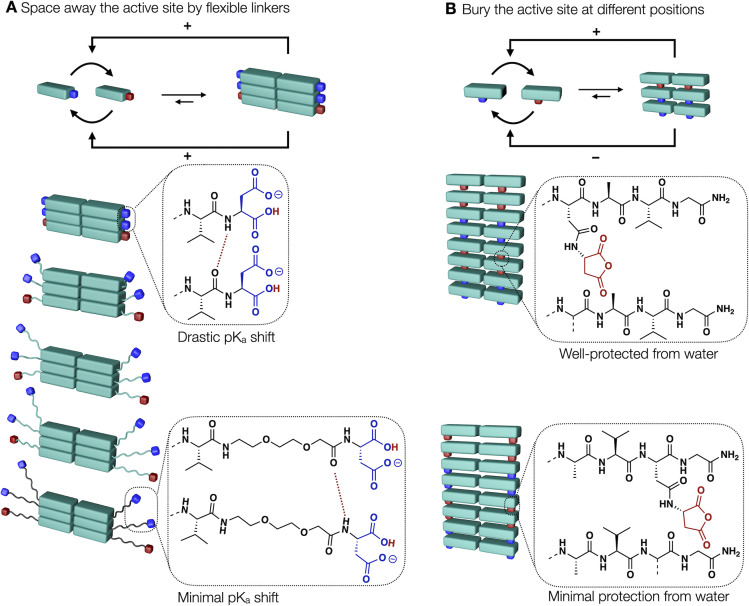
Simplified scheme of molecular design tailoring the nature of feedback and the reciprocal coupling strength between assembly and reaction cycle. (A) Strategy of spacing away the active site. (B) Strategy of burying the active site.

We establish the following design rules to regulate the reciprocal coupling between assembly and cycle. Molecular designs that yield high internal order in the assembly carried over to the active sites result in p*K*_a_ shift which changes the kinetics of the activation. This organization also yields a higher deactivation (Fig. 4). Spacing the active site away from the ordered assembly with a flexible linker decreases the effect (Fig. 4 and 2). Moreover, molecular designs in which the active site is buried in the hydrophobic fiber core, excludes it from water, and thus protects it from hydrolysis resulting in a strong deceleration of the deactivation reaction (Fig. 4 and 3).

## Conclusions

We studied two molecular design strategies to tune the nature of feedback and the degree of reciprocal coupling between the assembly and reaction cycle in chemically fueled peptide assemblies (fibers). The reciprocal coupling couples to the internal structure, *i.e.* the rigidity of the peptide assembly, which we can tune with molecular design. We show that we can tune the propensity of the peptide to form β-sheet hydrogen bonding by spacing the chemically active site away from the peptide core or burying it in the peptide core responsible for the fiber rigidity. These design strategies enable us to tune the degree and nature of feedback. Thus, our findings allow us to establish heuristic design rules for controlling the degree and nature of reciprocal feedback. We envision that these design rules can be exploited in future work to create synthetic assemblies with kinetic asymmetry and tunable dynamic behavior like pattern formation, oscillations in morphology, and motility, as seen in living systems.

## Data availability

The data supporting this study's findings are available from the corresponding author upon reasonable request.

## Author contributions

J. B., B. A. K. K., and X. C. conceived the research and wrote the manuscript. J. B., B. A. K. K., and X. C. designed the experiments and analyzed the data. A. M. B. performed Cryo-TEM measurements.

## Conflicts of interest

The authors declare no competing financial interest.

## Supplementary Material

SC-014-D3SC02062B-s001
