# Supplementary material for: Design rules for reciprocal coupling in chemically fueled assembly
Source: Chem Sci. 2023 Aug 22;14(37):10176–83. doi: 10.1039/d3sc02062b (PMC10530897; doi:10.1039/d3sc02062b)
Supplement: SC-014-D3SC02062B-s001 [file SC-014-D3SC02062B-s001.pdf]

Electronic Supplementary Material (ESI) for Chemical Science.
This journal is © The Royal Society of Chemistry 2023

Electronic supplementary information for:

Design rules for reciprocal coupling in chemically fueled assembly.

Xiaoyao Chen,‡[a] Brigitte A. K. Kriebisch,‡[a] Alexander M. Bergmann,[a] Job Boekhoven*[a]

[a] Department of Chemistry, School of Natural Sciences, Technical University of Munich, Lichtenbergstrasse 4, 85748 Garching bei München, Germany.

‡ These authors contributed equally to this work.

Experimental Procedures

Materials & Methods

Materials. Acetonitrile (ACN) (High-performance liquid chromatography (HPLC) grade), Benzylamine, Deuterated dimethyl sulfoxide (DMSO-d_6), Deuterium oxide (D_2O), *N, N'*-Diisopropylcarbodiimide (DIC), Diisopropylethylamine (DIPEA), Dichloromethane (DCM), *N, N*-Dimethylformamide (DMF), 1-Ethyl-3-(3-dimethylaminopropyl)carbodiimide hydrochloride (EDC · HCl), Ethyl (hydroxyimino)cyanoacetate (oxyma) (Novabiochem®), Fmoc-Asp(OtBu)-*Wang* resin (100-200 mesh, loading: 0.68 $mmolg^{-1}$), Hydroquinone, 37% Hydrochloric acid (HCl), 1-Hydroxybenzotriazol (HOBt), 3-[Bis(dimethylamino)methyliumyl]-3H-benzotriazol-1-oxide hexafluorophosphate (HBTU), Methanol (MeOH), 4-Morpholineethanesulfonic acid (MES) buffer, Nile Red, Piperidine (99%), protected amino acids ($Asp(OtBu)_2$ hydrochloride, Ac-F-OH, Fmoc-G-OH, Fmoc-A-OH, Fmoc-V-OH, Fmoc-8-Amino-3,6-dioxaoctanoic acid, Fmoc-Asp(OtBu)-OH), Kaiser test kit, Rink amide resin (100-200 mesh, loading: 0.74 $mmolg^{-1}$), Sodium hydroxide (NaOH), Thioflavin T (ThT), Trifluoroacetic acid (99%, TFA), and Triisopropylsilane (TIPS) were purchased from Sigma-Aldrich unless indicated otherwise. All peptides were synthesized using standard fluoren-9-ylmethoxycarbonyl (Fmoc) solid-phase peptide synthesis (SPPS) on Wang resin or Rink amide resin (see methods - Peptide synthesis and purification). All chemicals were used without any further purification unless indicated otherwise.

Methods

Peptide synthesis procedure 1. Ac-FAVD, Fmoc-AVG_nD (n = 0, 1, 2, 3), and Fmoc-DAVG-NH_2, were synthesized on a 0.5 mmol scale using a CEM Liberty microwave-assisted peptide synthesizer, and the Liberty Blue Application Software (Copyright© CEM Corporation 2015, Version: 1.45.5794.20265). For Ac-FAVD and Fmoc-AVG_nD preloaded Wang resin (Fmoc-Asp(OtBu)-Wang) (100-200 mesh, loading: 0.68 $mmolg^{-1}$) was used. For Fmoc-DAVG-NH_2, Rink amide resin (100-200 mesh, loading: 0.74 $mmolg^{-1}$) was used. The respective resin was swelled for 30 minutes in 10 mL of DMF. Then a 20% piperidine (2×10 mL) solution in DMF was used to remove the *N*-terminal Fmoc protecting group by heating in the microwave (1×1 minute, 90 °C). DMF (2×10 mL) was added to wash the reaction mixture. The first amino acid coupling was achieved by using 4 equivalents (eq.) of amino acid in DMF (0.2 M, 10 mL), 4 eq. of DIC (0.5 M, 4 mL), and 4 eq. of the oxyma (1 M, 2 mL). Next, the resin solution was heated in the microwave (1×2 minutes, 90 ºC). The coupling was repeated twice to increase the yield. The Fmoc-deprotection, washing, 2 × couplings, washing cycle was carried out for every amino acid coupling until the last amino acid of the respective peptide sequence was coupled. Peptides were cleaved from the resin using a mixture (10 mL) of 95% TFA (99%), 2.5% MQ water, and 2.5% TIPS. After removing TFA, purification on reversed-phase (RP)-HPLC and lyophilizing were conducted to get the pure peptides.

Peptide synthesis procedure 2. Fmoc-AVGlycolD, Fmoc-ADVG-NH_2, and Fmoc-AVDG-NH_2 were synthesized from N- to C-terminus on a 0.5 mmol scale following the given synthesis protocol. For Fmoc-AVGlycolD preloaded Wang resin (FmocAsp(OtBu)-Wang, (100-200 mesh, loading: 0.68 mmolg-1)) was used, and for Fmoc-ADVG-NH_2 and Fmoc-AVDG-NH_2 Rink amide resin (100-200 mesh, loading: 0.74 $mmolg^{-1}$) was used. The respective resin was swelled for 30 minutes in 10 mL of DMF at room temperature and transferred to a reaction vessel that was connected via a three-way cock to a waste container-water pump for draining the reaction vessel and to an N_2-flow for agitation. The solid-phase peptide synthesis was performed at 68°C by placing the reaction vessel in a silicon oil bath. First, the resin was swelled for 5 minutes at 68°C in 10 mL DMF under continuous agitation by N_2-bubbling. Then DMF was removed via draining, and 10 mL mixture of 5% (volume percentage) piperazine with 0.2 M HOBt in DMF was added and agitated for one minute. The piperazine/HOBt was removed *via* draining. Subsequently, 20 mL of 5% piperazine/0.2 M HOBt in DMF was added and agitated for 5 minutes. The resin was washed 4x with 10 mL DMF before adding 3 eq/r amino acid, 3 eq/r HCTU, 6 eq/r DIPEA in 4 mL DMF to the resin and agitating the reaction mixture for 6 minutes. The reaction solution was drained, and the resin was washed 4x with 10 mL DMF. The Fmoc-deprotection, washing, amino acid coupling, washing cycle was repeated for every amino acid until the last amino acid was coupled. Fmoc-AVGlycolD, Fmoc-ADVG-NH_2, and Fmoc-AVDG-NH_2 was cleaved from the resin using a mixture (10 mL) of 95% TFA (99%), 2.5% MQ-water and 2.5% TIPS and dried in vacuo.

Peptide synthesis procedure for Fmoc-D(D)AVG-NH_2, Fmoc-AD(D)VG-NH_2 and Fmoc-AVD(D)G-NH_2

Fmoc-D(D)AVG-NH_2, Fmoc-AD(D)VG-NH_2 and Fmoc-AVD(D)G-NH_2 were synthesized by coupling $Asp(OtBu)_2$ to the purified Fmoc-DAVG-NH_2 and the non-purified Fmoc-AD(D)VG-NH_2 and Fmoc-AVD(D)G-NH_2. Fmoc-DAVG-NH_2 was dissolved in 10 mL DMF. HOBt (3 eq.) and HBTU (3 eq.) and DIPEA (7 eq.) were added under N_2 at room temperature with stirring for 5 minutes. Di-tert-butyl L-Aspartate Hydrochloride 98.0+% (3 eq.) was added and stirred overnight. After removing DMF under vacuum, a mixture (10 mL) of 95% TFA (99%), 2.5% MQ-water, and 2.5% TIPS was added to cleave the tert-butyl protecting group. After removing TFA, purification on RP-HPLC and lyophilizing was conducted to get the pure peptide.

Peptide purification. Crude peptides were purified using preparative reversed-phase HPLC (Thermofisher Dionex Ultimate 3000, Hypersil Gold 250x4.8 mm, Thermo Scientific™ Dionex™ Chromelon™ Chromatography Data System, Version: 7.2SR4(8179), Copyright© 2009 – 2016 Thermo Fisher Scientific Inc.). For Fmoc-AVG$_n$D, Fmoc-AVGlycolD, Fmoc-DAVG-NH_2, Fmoc-D(D)AVG-NH_2, Fmoc-AD(D)VG-NH_2 and Fmoc-AVD(D)G-NH_2, a linear gradient of water/acetonitrile (each with 0.1% TFA) from 60:40 to 2:98 was used, detecting at 254 nm with a flow of 20 mL min^{-1}. For Ac-FAVD, a linear gradient of water/acetonitrile (each with 0.1% TFA) was used from 98:2 to 2:98 with detection at 220 nm. The purified peptides were lyophilized (Lyophylle: Alpha LDplus, Christ) and stored at -20 °C until further use. The purity of the peptides was analyzed by ^{1}H-NMR spectroscopy (Fig. S20-24), Electrospray Ionization Mass Spectrometry in positive mode (ESI-MS; Table S1), as well as analytical HPLC (Thermofisher Dionex Ultimate 3000, eluted with a linear gradient of Water/ACN (each with 0.1% TFA) from 98:2 to 2:98, Fig. S3-4, S 16).

General sample preparation. We used the following standard conditions for all experiments: 2.5 mM Ac-FAVD, G$_n$, Glycol peptide (GY), and D(D)$_n$ in 200 mM MES buffered water, pH 6 at 21 °C, and 50/25/10 mM EDC.

Stock solutions of the peptide precursor (2.5 mM) were prepared in 200 mM MES buffer at pH 6. The pH of the peptide stocks was adjusted to pH 6. For all experiments, 2 M or 3.5 M EDC stock solutions were prepared freshly in 200 mM MES buffer at pH 6. Reaction cycles were started by adding EDC to the peptide stock at 21 °C. 400 mM benzylamine stocks were prepared freshly in acetonitrile. 200 mM MES buffered water was prepared by dissolving the MES hydrate in MQ water. The pH of the 200 mM MES buffered water was adjusted to pH 6, using 1 M sodium hydroxide solution.

^{1}H-NMR spectra were conducted on a Varian Inova 300 (300 MHz) and Varian Inova 500 (500 MHz). Chemical shifts are reported as δ-values in parts per million (ppm) relative to the internal standard hydroquinone (50 mM) in D_2O (δH: 6.70) or the deuterated solvent peak: DMSO-d_6 (δH: 2.50).

^{1}H-NMR spectroscopy (300 MHz) was used to determine the assembled peptide concentration during the reaction cycle. An inner tube with an internal hydroquinone standard (50 mM) dissolved in D_2O was used. The ^{1}H-NMR spectra were recorded with water suppression. The non-assembled peptide concentration was determined by comparing the integral of the Fmoc-protons at 7.20 – 7.50 ppm to the integral of the internal hydroquinone standard at 6.70 ppm. MestReNova© software (Version 11.0.0.-17609) was used to analyze all recorded NMR spectra.

We determined the fiber composition using NMR spectroscopy. Here, we use the phenomenon that non-assembled molecules are NMR-visible while self-assembled molecules are NMR-silent. The NMR-silencing of assembled molecules is based on the reduced transversal relaxation time (T2) from molecules in a self-assembled state leading to signal-broadening. When we fuel 2.5 mM G$_n$, Glycol peptide, D(D)$_n$ with 50 mM EDC, most of the precursor and the activated product were not visible by ^{1}H-NMR spectroscopy in the first 30 minutes of the reaction cycle. This result suggests that most peptides, whether in the precursor or activated state, were in the assembled state (Fig. 2E, 3 D, Fig. S 9, S 10, S 15, S 19). By combining the NMR data with the kinetic data from HPLC, we calculated the concentration of precursor in the assembly using the following equation:

$[\text{precursor}]_{\text{in assembly}} = [\text{peptide}]_{\text{in assembly}} - [\text{product}]$

Here, we assume that all product is assembled.

Analytical reversed-phase HPLC (ThermoFisher Vanquish Duo UHPLC, a Hypersil Gold 100 x 2.1 mm C18 column (3 µm pore size)) was used to determine the concentration profile of EDC, precursor, and anhydride (product) over time. We used the quenching method described by Schnitter and Boekhoven (2020)[1] to indirectly determine the concentration of the anhydride by converting the anhydride irreversibly into a monoamide, which we refer to as benzylamide. Briefly, after starting the reaction cycle by adding EDC to the peptide precursor solution with a total volume of 10 µL, we added 10 µL of 400 mM benzylamine at certain time points. The resulting clear solution (pH > 9) was then measured *via* HPLC to determine the concentration of EDC, peptide, and anhydride, *i.e.* benzylamide. All compounds involved were separated using a linear gradient of ACN (2% to 98%) and water with 0.1% TFA. Measurements were performed at 8 °C to slow down the Fmoc cleavage under the respective basic reaction conditions. Calibration curves for EDC (λ = 220 nm), the precursor (λ = 254 nm) and benzylamine (λ = 254 nm) were obtained to quantify these compounds over time (Fig. S 25). To determine the benzylamide concentration, *i.e.*, the anhydride concentration, we calculated the calibration value from the respective peptide and benzylamine calibration value at λ = 254 nm. All experiments were performed in triplicate (n = 3). Retention times and calibration values are given in Supporting Table S1, S2, and Fig. S 3-4, 16, 25.

Titration. For 10 mM peptide concentration, the required amount of peptide for 200 µL total volume was first suspended into 100 µL of MQ-water. NaOH (0.1 M) was added to the aqueous peptide suspensions until pH > 10 was reached. The volume was adjusted to 200 µL. The samples were vortexed to dissolve the peptide. The titration was performed by stepwise addition of small volumes of 0.1 M HCl at 25 °C. After each addition of HCl, the samples were vortexed. The pH was measured with a HI 2210 pH meter from HANNA® Instruments. The titration curves were analyzed with the Hyperquad2008 program[2] to obtain the respective pK_a of the peptide precursor.

Electron Spray Ionization – Mass Spectrometry (ESI-MS). ESI-MS experiments were conducted on LCQ Fleet Ion Trap Mass Spectrometer (Thermo Scientific). All samples were measured in positive mode and analysis was performed using the Thermo Xcalibur Qual Browser 2.2 SP1.48 software LCQ Fleet Ion Trap Mass Spectrometer. 1 µL of the samples were directly injected into an analytical reversed-phase C18 column upstream of the 3D ion trap (LCQ Fleet Ion Trap Mass Spectrometer) without further dilution. The samples were tracked by mass in positive mode with a UV/Vis detector at 220 nm, and 280 nm. All compounds involved were separated using linear gradients of water (A) and acetonitrile (B) from 95:5 to 5:95 in 8 min and applying a flow rate of 0.7 $mLmin^{-1}$. Both eluents contained 0.1% formic acid.

Confocal Fluorescence Microscopy. Confocal fluorescence microscopy was performed on a Leica TCS SP8 confocal microscope using a 63x water immersion objective with a numerical aperture of 1.2.
Samples with a total reaction volume of 20 µL were prepared directly in an ibidi µ-slide angiogenesis well plate. 2.5 µM Nile red was added as a dye with excitation at 552 nm and emission at 577 – 656 nm. A laser intensity of 0.3% and a pinhole of 111.3 µm ($\lambda_{emission}$ = 580 nm), was used to image the samples. ImageJ 1.52p (Java 1.80_172, 64-bit) was used to analyze all recorded images. Imaging was conducted at 22 °C.

Fluoresecence plate reader.
Thioflavin T (ThT) assay: 2.5 µM ThT was added to the reaction solution of 2.5 mM peptide with 10 mM EDC.
The fluorescence intensities were measured every minute at an emission wavelength of 485 nm and excitation at 440 nm over time. The excitation and emission slit width was 12 nm. The ThT spectra were recorded from 460 nm – 600 nm. The excitation and emission slit width was 5 nm.
Nile Red assay: 2.5 µM Nile Red was added to the reaction solution of 0/0.25/0.5/0.75 mM peptide with 50 mM EDC. The fluorescence intensities were measured every two minutes at an emission wavelength of 630 nm and excitation at 530 nm over time. The excitation and emission slit width was 12 nm.

Fourier-Transform Infrared Spectroscopy (FT-IR). FT-IR spectra were obtained of the self-assemblies formed by G_n, Glycol, $D(D)_n$ peptides 3 minutes after EDC addition. For all samples, 2.5 mM peptide was fueled with 50 mM EDC in 200 mM MES in D_2O, pH 6. Samples were freshly prepared on the measurement crystal of the Elmer Perkin Frontier FT-IR. The transmittance of the samples was measured from 4000 cm^{-1} to 650 cm^{-1}. 16 scans were conducted per measurement. FT-IR spectra were cut between 1550 cm^{-1} and 1750 cm^{-1}.
For the FT-IR measurement, peptide stocks, EDC, and MES buffer were prepared in D_2O as described in the general sample preparation.

Cryogenic-Transmission Electron Microscopy (Cryo-TEM). Cryo-TEM imaging was performed on a Tecnai Spirit microscope (FEI/Thermo Fisher) operating at 120 kV. Images were conducted in a low-dose mode on a CCD camera. Before use, Cu-grids (C-flat, 2.0 µm hole size, 2.0 µm hole spacing, 400 mesh) were freshly glow discharged for 90 seconds at 45 mA and 3 × 10-2 mbar. Samples of 50 µL reaction volume were freshly prepared for Cryo-TEM as described above. Aliquots of 5 µL were transferred onto the Cu-grids in an FEI/Thermo Fisher Vitrobot with 30 seconds waiting time, a plot time of 2.5 seconds, and a plot force of -1. The sample was set to a relative humidity of 100% at 22 °C. Thereafter, the grids were plunged into liquid ethane that was pre-cooled by liquid nitrogen. The cryo-TEM grids were stored in liquid nitrogen until imaging. For imaging, the cryo-TEM grids were placed into a Gatan cryo-transfer-specimen holder. The temperature of the Gatan cryo-transfer-specimen holder was maintained at -170°C during the whole process.

Kinetic Model

We used a kinetic model written in Python to model the reaction cycle. Details can be found in our recent work from Chen *et al.*[3, 4] and Hartly's work.[5] The kinetic model calculates the concentration of all relevant components over time in the reaction cycle based on five differential equations, which describe five chemical reactions: 1) direct hydration of EDC (r0), 2) the activation of acid (r1), 3) the attack of the O-acylisourea by the second intramolecular carboxylic acid to form the anhydride (r2), 4) the hydrolysis of the O-acylisourea (r3), and 5) the hydrolysis of the anhydride (r4). The kinetic model does not include the side reaction: The formation of N-acylisourea *via* the N to O shift of the O-acylisourea, as N-acylisourea is not formed and observed in the reaction cycle where aspartic acid derivates are used as a precursor.[4, 6] The fundamental reactions of the kinetic model are described below.

All the k-values were free parameters to optimize. Because O-acylisourea cannot be observed, the individual values of k_2 and k_3 cannot be accurately extracted. However, their ratio appears *i.e.* that $K = k_3/k_2 << 1$, in line with an intramolecular process. Noteworthy, the system could also be successfully fitted even with a simpler model that depends only on k_1 and k_4, indicating that all the EDC reacting with the aspartic acid is used to form the anhydride. All the rate constant values are listed in Table S3.

1) Reaction 0 (k_0): The direct hydration of carbodiimide (EDC) with a first-order rate constant.
($R_1 = C_2H_5$, $R_2 = C_4H_{12}N$)

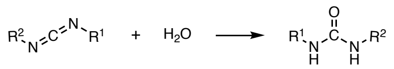

2) Reaction 1 (k_1): The reaction of carbodiimide with the precursor to O-acylisourea with a second-order rate constant.
(R = respective peptide chain)

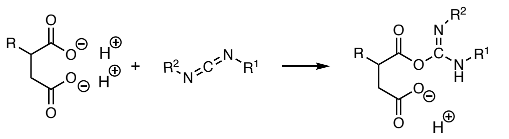

3) Reaction 2 (k_2): The formation of anhydride with a first-order rate constant.

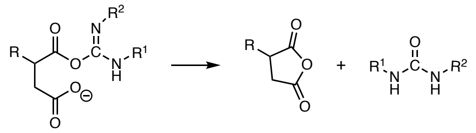

4) Reaction 3 (k_3): Direct hydrolysis of O-acylisourea with a first-order rate constant.

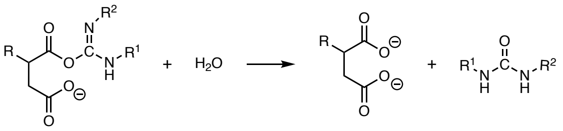

5) Reaction 4 (k_4): The reaction describes the hydrolysis of the anhydride and takes place with a (pseudo)-first order rate.

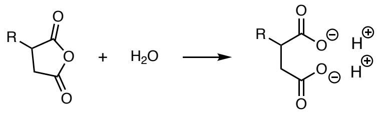

Supporting Tables

***Table S1:** Characterization of synthesized peptide precursors.*

Name	Structure	Exact Mass (gmol^{-1})	Mass found (gmol^{-1})	Calibration value with 1 uL injection, 254nm
Ac-FAVD	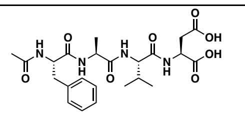	M_w = 492.22<br>$C_{23}H_{32}N_4O_8$	492.91<br>[M_w+H^+]	0.4925
G_0	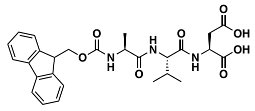	M_w = 525.21<br>$C_{27}H_{31}N_3O_8$	525.97<br>[M_w+H^+]	33.462
G_1	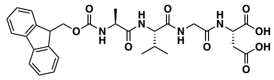	M_w = 582.23<br>$C_{29}H_{34}N_4O_9$	582.94<br>[M_w+H^+]	30.972
G_2	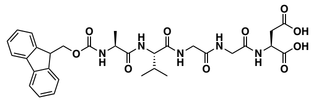	M_w = 639.25<br>$C_{31}H_{37}N_5O_{10}$	640.06<br>[M_w+H^+]	31.631
G_3	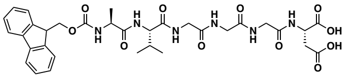	M_w = 696.28<br>$C_{33}H_{40}N_6O_{11}$	697.08<br>[M_w+H^+]	30.626
Glycol	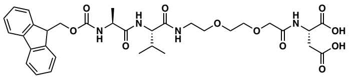	M_w = 670.29<br>$C_{33}H_{42}N_4O_{11}$	671.21<br>[M_w+H^+]	30.266
$D(D)_1$	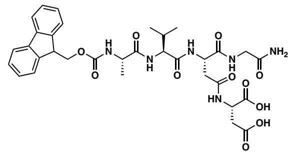	M_w = 696.28<br>$C_{33}H_{40}N_6O_{11}$	697.15<br>[M_w+H^+]	30.510
$D(D)_2$	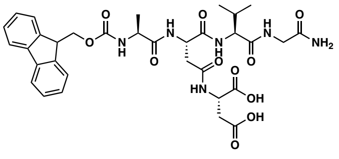	M_w = 696.28<br>$C_{33}H_{40}N_6O_{11}$	697.05<br>[M_w+H^+]	30.072
$D(D)_3$	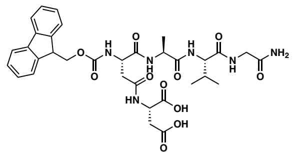	M_w = 696.28<br>$C_{33}H_{40}N_6O_{11}$	696.91<br>[M_w+H^+]	29.121
EDC・HCl	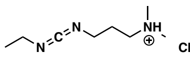	-	-	16.336 (220nm)
Benzylamine	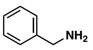	-	-	0.4426

Table S2: *Product characterization of the benzylamine quenched chemical reaction cycle.*

**Name (purity)**	**Structure**	**Exact Mass ($gmol^{-1}$)**	**Mass found ($gmol^{-1}$)**
Ac-FAVD-benzylamide	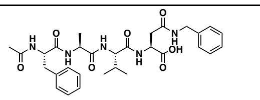	M_w = 581.28<br>$C_{30}H_{39}N_5O_7$	581.99<br>$[M_w+H^+]$
	Two dimers but only show one, same as below		
G_0-benzylamide	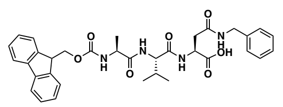	M_w = 614.27<br>$C_{34}H_{38}N_4O_7$	615.05<br>$[M_w+H^+]$
G_1-benzylamide	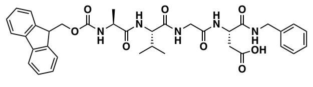	M_w = 671.3<br>$C_{36}H_{41}N_5O_8$	672.02<br>$[M_w+H^+]$
G_2-benzylamide	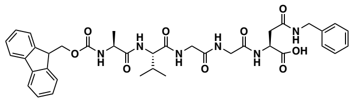	M_w = 728.32<br>$C_{38}H_{44}N_6O_9$	729.09<br>$[M_w+H^+]$
G_3-benzylamide	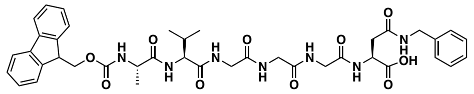	M_w = 785.34<br>$C_{40}H_{47}N_7O_{10}$	786.14<br>$[M_w+H^+]$
Glycol-benzylamide	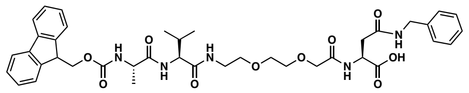	M_w = 759.35<br>$C_{40}H_{47}N_7O_{10}$	760.28<br>$[M_w+H^+]$
$D(D)_1$-benzylamide	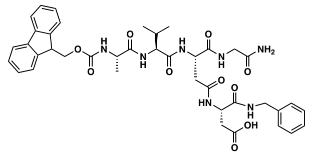	M_w = 785.34<br>$C_{40}H_{47}N_7O_{10}$	786.25<br>$[M_w+H^+]$
$D(D)_2$-benzylamide	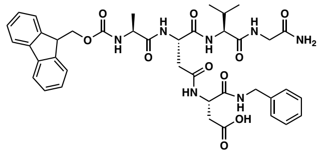	M_w = 785.34<br>$C_{40}H_{47}N_7O_{10}$	786.14<br>$[M_w+H^+]$
$D(D)_3$-benzylamide	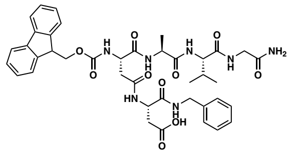	M_w = 785.34<br>$C_{40}H_{47}N_7O_{10}$	786.06<br>$[M_w+H^+]$

Table S3*: Rate constant used in the kinetic model at pH 6 fueled with EDC.*

	**k_0 (min^{-1})**	**k_1 ($mM^{-1}min^{-1}$)**	**K**	**k_4 (min^{-1})**	**Half life of anhydride (calculated by ln(2)/k_4)**
Order	1st	2nd	1st	1st	
Ac-FAVD	1.84×10^{-4}	0.007	0	0.6	1.2 min
G_0	1.84×10^{-4}	0.0301	2.34×10^{-8}	1.23	0.6 min
G_1	1.84×10^{-4}	0.0228	0	1.3	0.5 min
G_2	1.84×10^{-4}	0.0205	4.03×10^{-13}	1.24	0.6 min
G_3	1.84×10^{-4}	0.0188	7.03×10^{-12}	1.29	0.5 min
Glycol	1.84×10^{-4}	0.0150	0	0.90	0.8 min
$D(D)_1$	1.84×10^{-4}	0.0138	0	0.41	1.7 min
$D(D)_2$	1.84×10^{-4}	0.0125	0	0.40	1.7 min
$D(D)_3$	1.84×10^{-4}	0.0070	0	0.19	3.7 min

Supporting Figures

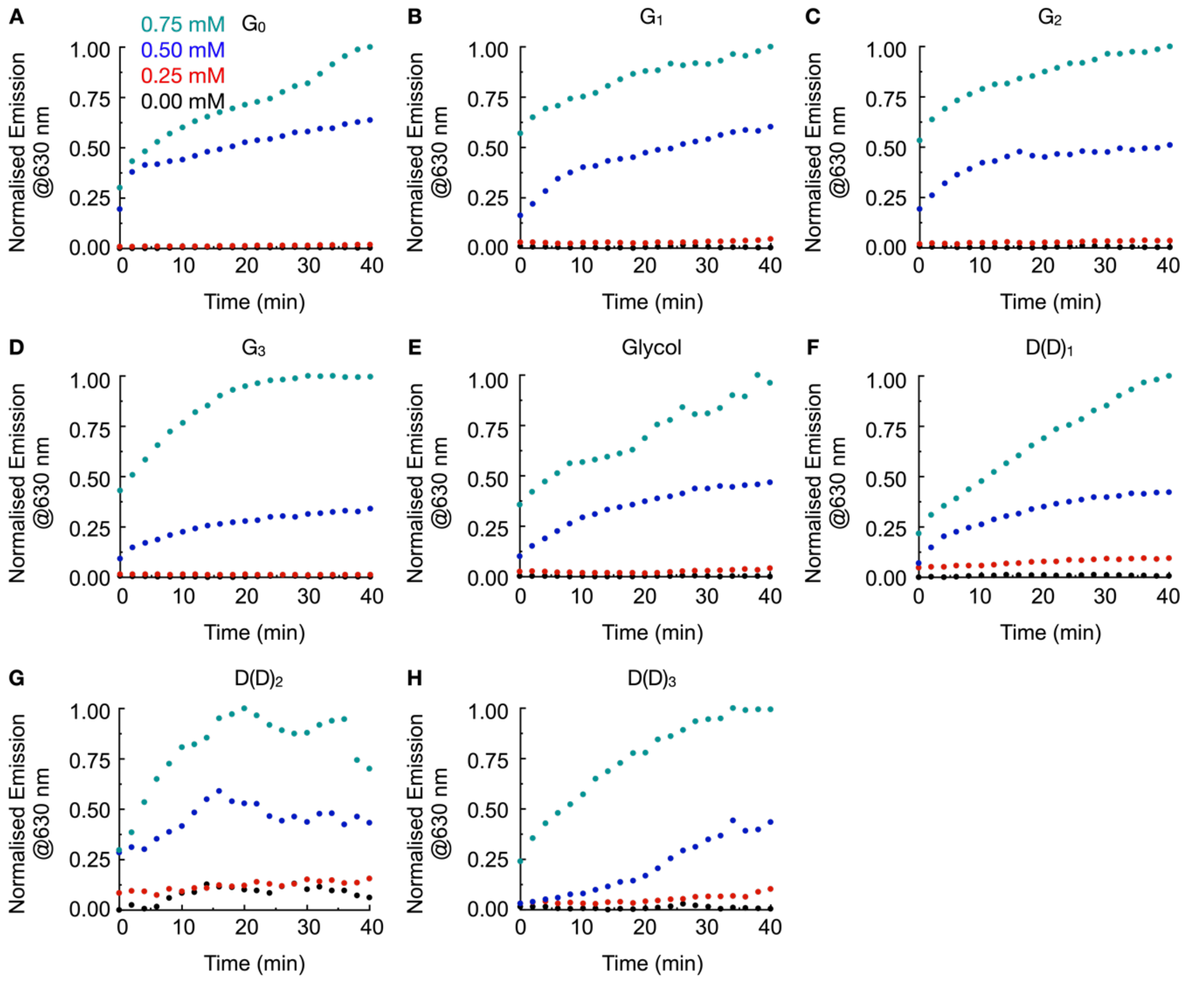

Figure S1: Nile Red fluorescence intensity at 630 nm after excitation at 530 nm against time of 0/0.25/0.5/0.75 mM peptide fueled with 50 mM EDC as a measure of the critical aggregation concentration of **A)** G_0 **B)** G_1 **C)** G_2 **D)** G_3 **E)** Glycol **F)** $D(D)_1$ **G)** $D(D)_2$ **H)** $D(D)_3$ in 200 mM MES buffer, pH 6.

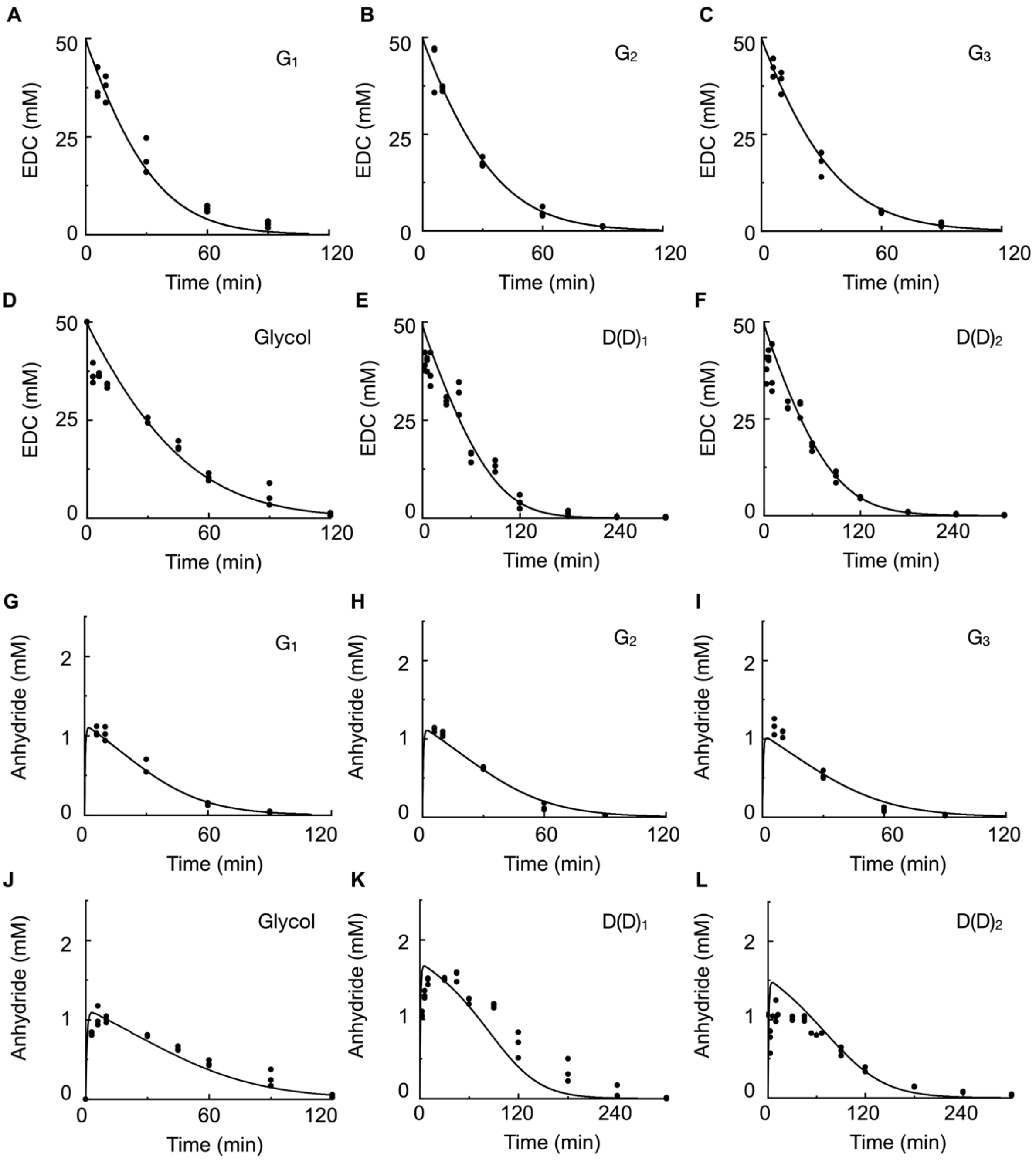

Figure S2: The EDC consumption as a function of time for 2.5 mM **A)** G_1 **B)** G_2 **C)** G_3 **D)** Glycol **E)** $D(D)_1$ **F)** $D(D)_2$ fueled with 50 mM EDC, in 200 mM MES buffer, pH 6. Anhydride concentration of **G)** G_1 **H)** G_2 **I)** G_3 **J)** Glycol **K)** $D(D)_1$ **L)** $D(D)_2$ as a function of time after fueling with EDC. HPLC (markers) and kinetic model (solid line), n = 3.

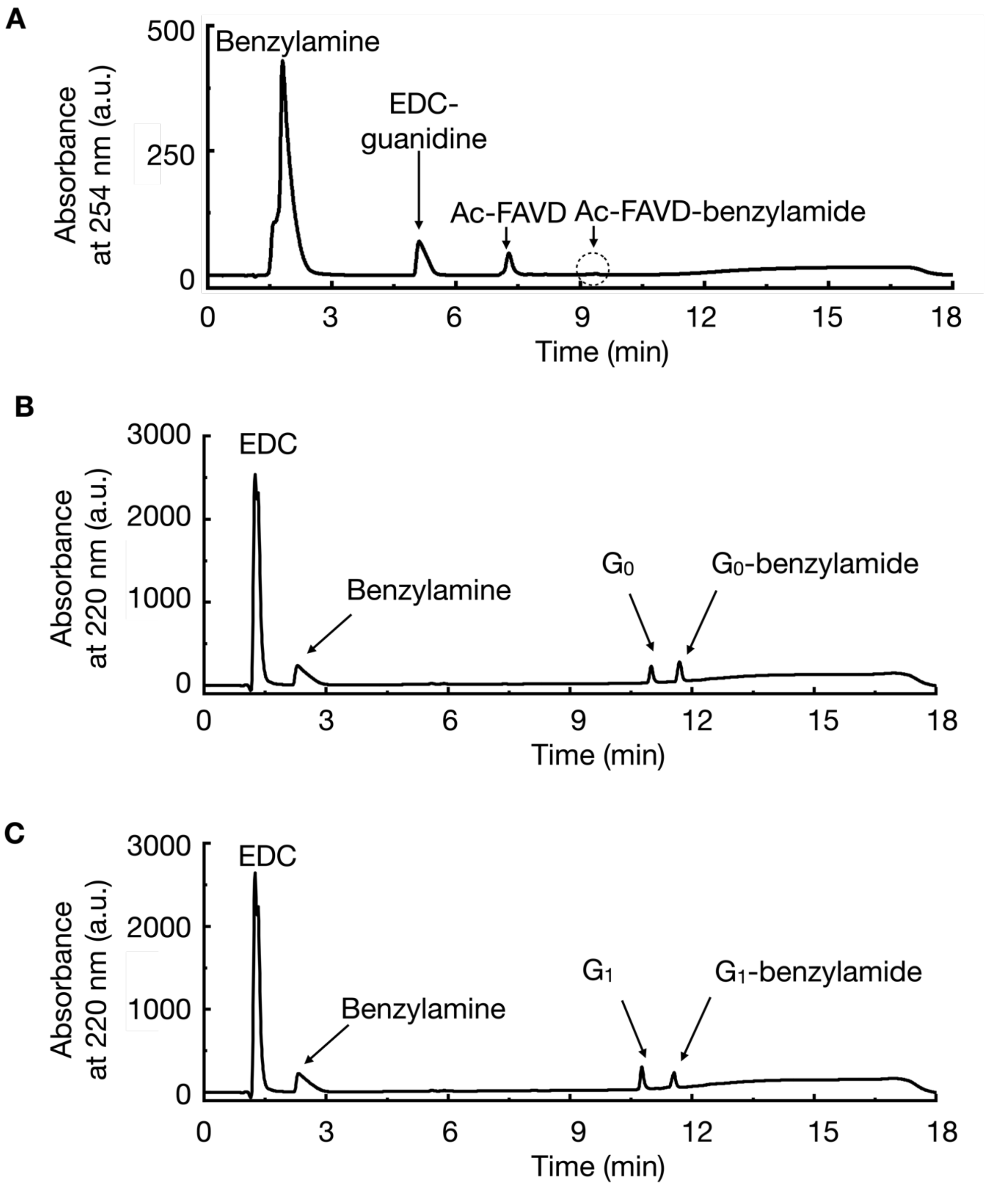

Figure S3: Representative HPLC-chromatogram at 3 min at 220 nm (injection volume: 1 µL) of 2.5 mM **A)** Ac-FAVD (254 nm, 2 µL injection volume), **B)** G_0, **C)** G_1 quenched 1:1 with 400 mM benzylamine at 3 min after adding 50 mM EDC.

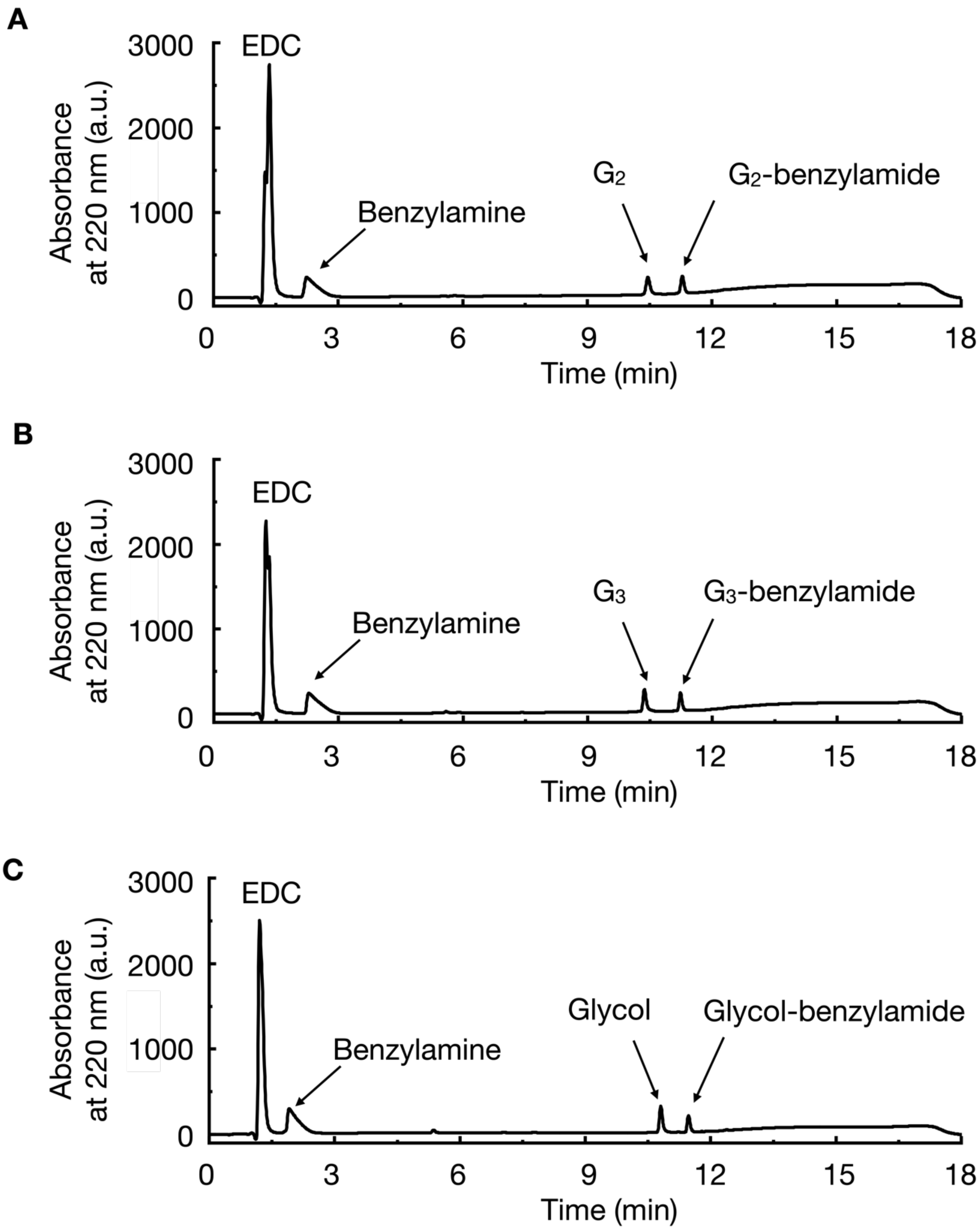

Figure S4: Representative HPLC-chromatogram at 3 min at 220 nm (injection volume: 1 μL) of 2.5 mM **A)** G_2, **B)** G_3, **C)** Glycol peptide quenched 1:1 with 400 mM benzylamine after adding 50 mM EDC.

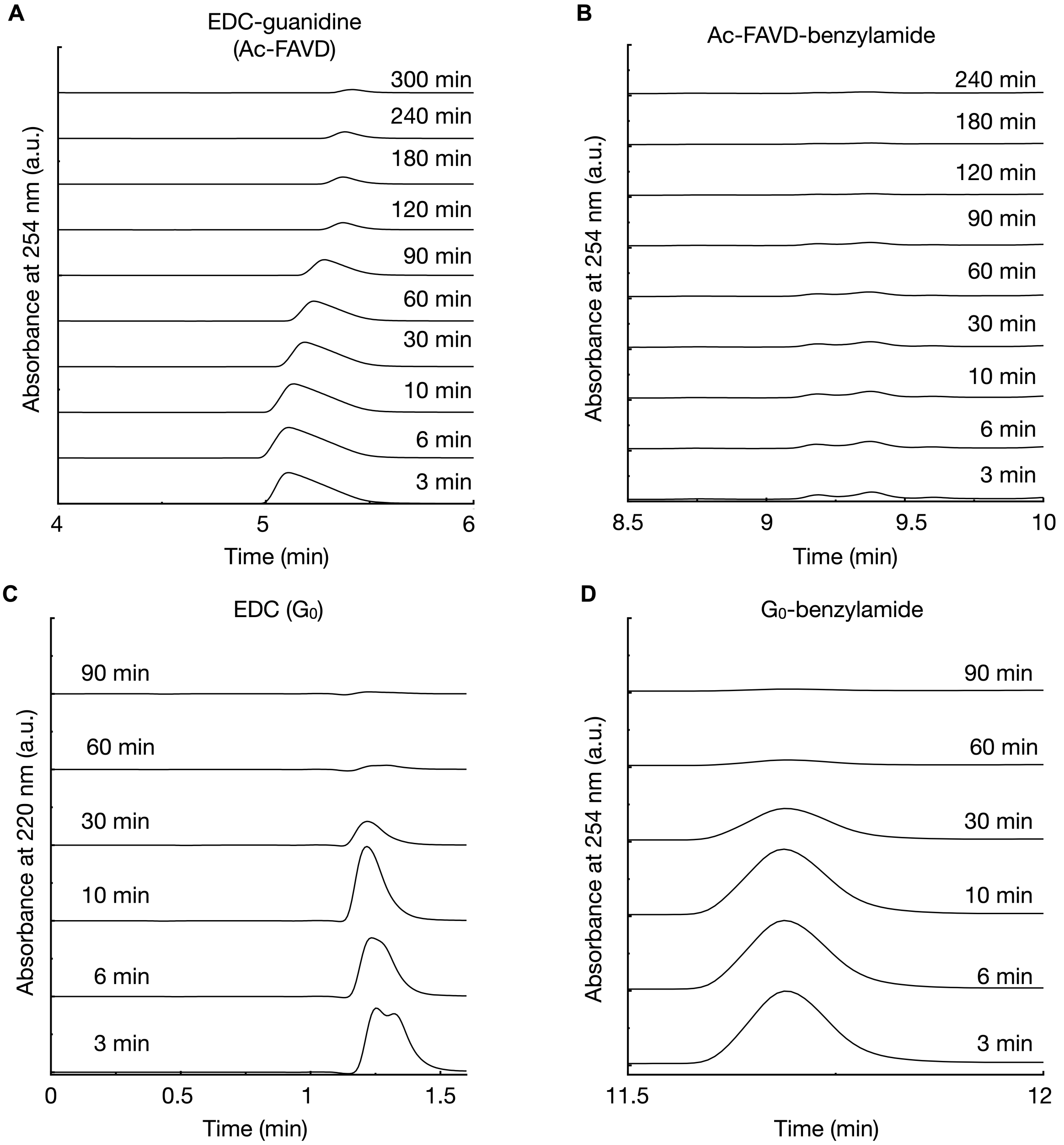

Figure S5: Representative HPLC-chromatograms of EDC-peak over time at 220 nm and benzylamide peak over time at 254 nm (injection volume: 1 µL) of 2.5 mM **A-B)** Ac-FAVD (2 µL injection volume, EDC guanidine at 254 nm), **C-D)** G_0 quenched 1:1 with 400 mM benzylamine after adding 50 mM EDC.

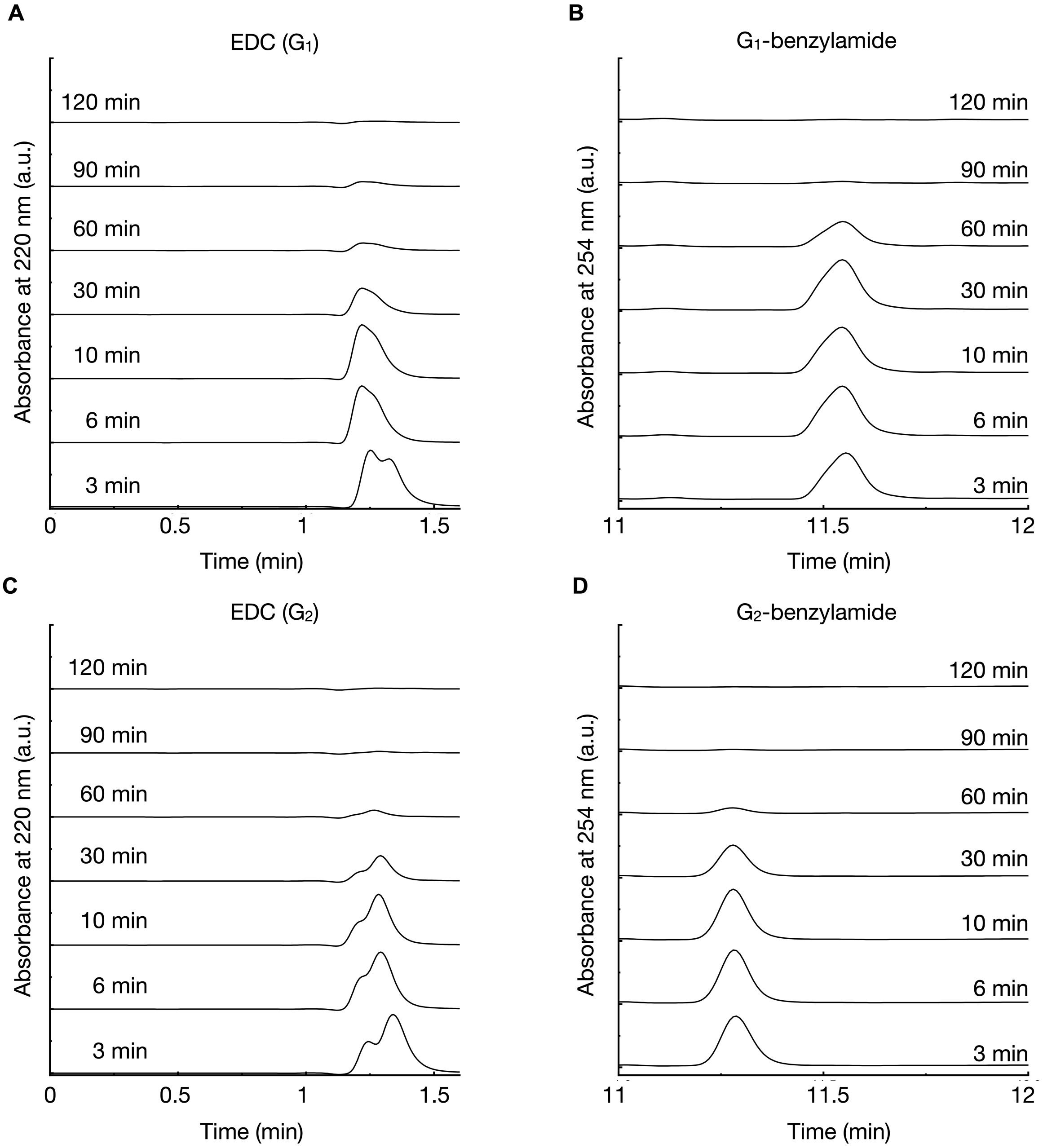

Figure S6: Representative HPLC-chromatograms of EDC-peak over time at 220 nm and benzylamide peak over time at 254 nm (injection volume: 1 µL) of 2.5 mM **A-B)** G_1, **C-D)** G_2 quenched 1:1 with 400 mM benzylamine after adding 50 mM EDC.

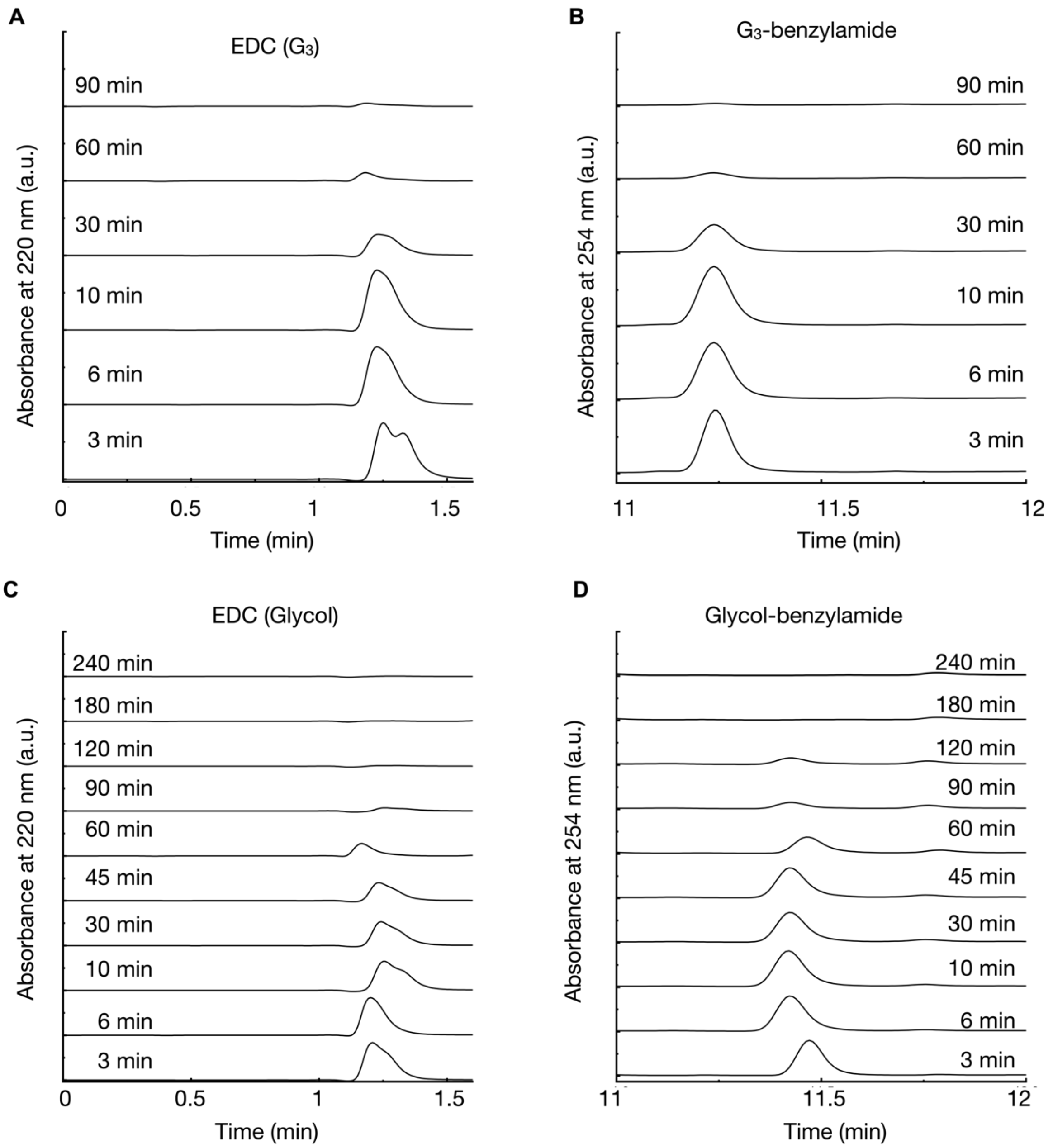

Figure S7: Representative HPLC-chromatograms of EDC-peak over time at 220 nm and benzylamide peak over time at 254 nm (injection volume: 1 µL) of 2.5 mM **A-B)** G_3, **C-D)** Glycol peptide quenched 1:1 with 400 mM benzylamine after adding 50 mM EDC.

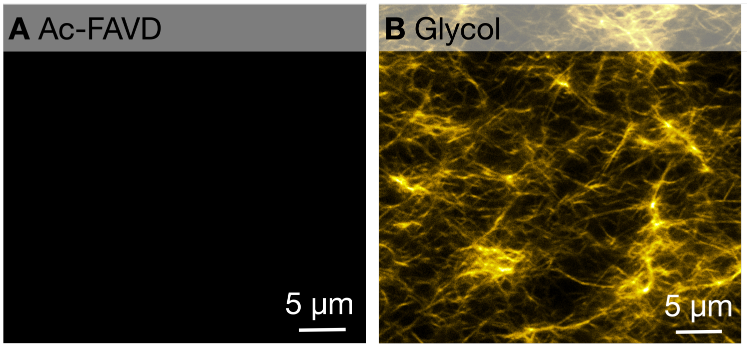

Figure S8: Confocal micrograph of **A)** Ac-FAVD and **B)** Glycol 10 minutes after fueling with EDC. Scale bar 5 μm.

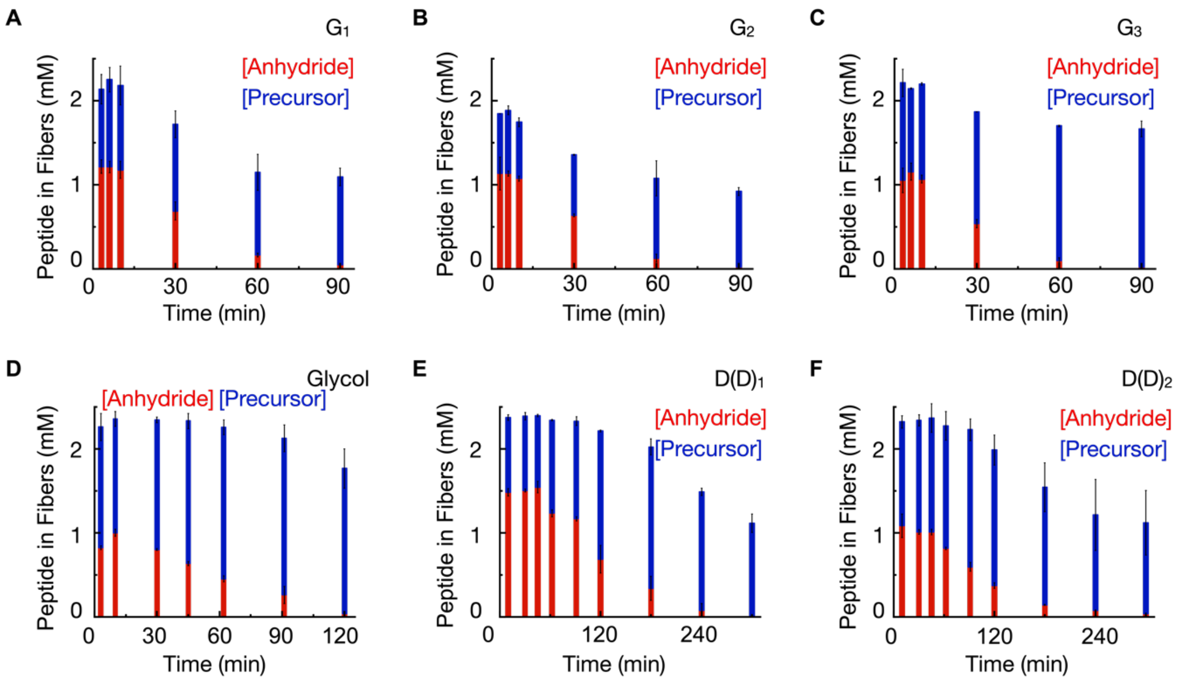

Figure S9: The concentration of precursor and anhydride product in the assemblies as a function of time. The peptide concentration in the assembly was determined by ^{1}H-NMR spectroscopy. The amount of anhydride product was determined by HPLC. 2.5 mM **A)** G_1 **B)** G_2 **C)** G_3 **D)** Glycol **E)** $D(D)_1$ **F)** $D(D)_2$ fueled with 50 mM EDC, in 200 mM MES buffer, pH 6. Experiments were done in duplicate.

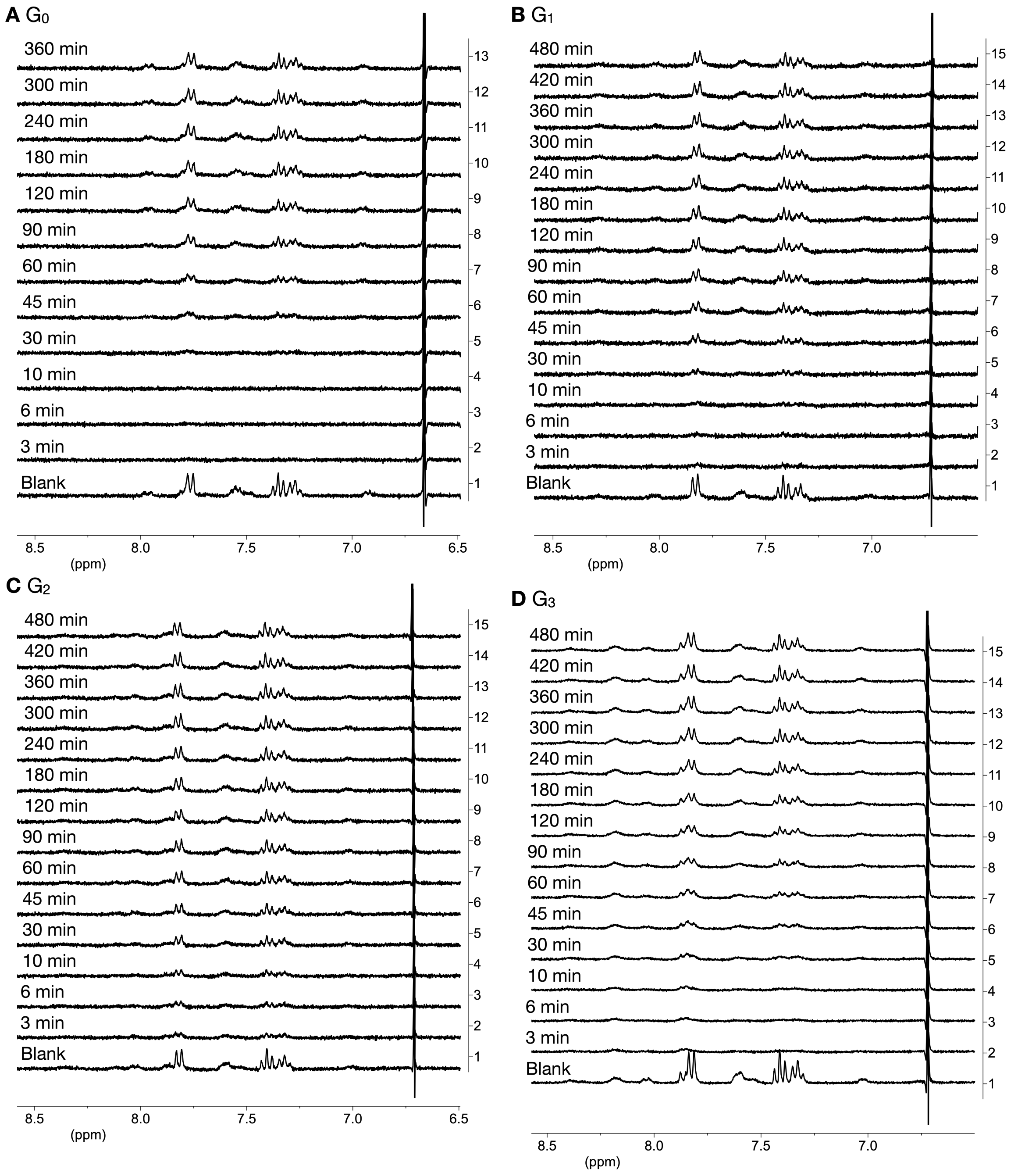

Figure S10: Representative ^{1}H-NMR spectra from 8.50 ppm to 6.50 ppm of 2.5 mM peptide fueled with 50 mM EDC as a function of time of **A)** G_0 **B)** G_1 **C)** G_2 **D)** G_3 in 200 mM MES, pH6, 300 MHz. The non-assembled peptide concentration was determined by comparing the integral of the Fmoc-protons at 7.20 – 7.50 ppm to the integral of the internal hydroquinone standard at 6.70 ppm. MestReNova© software.

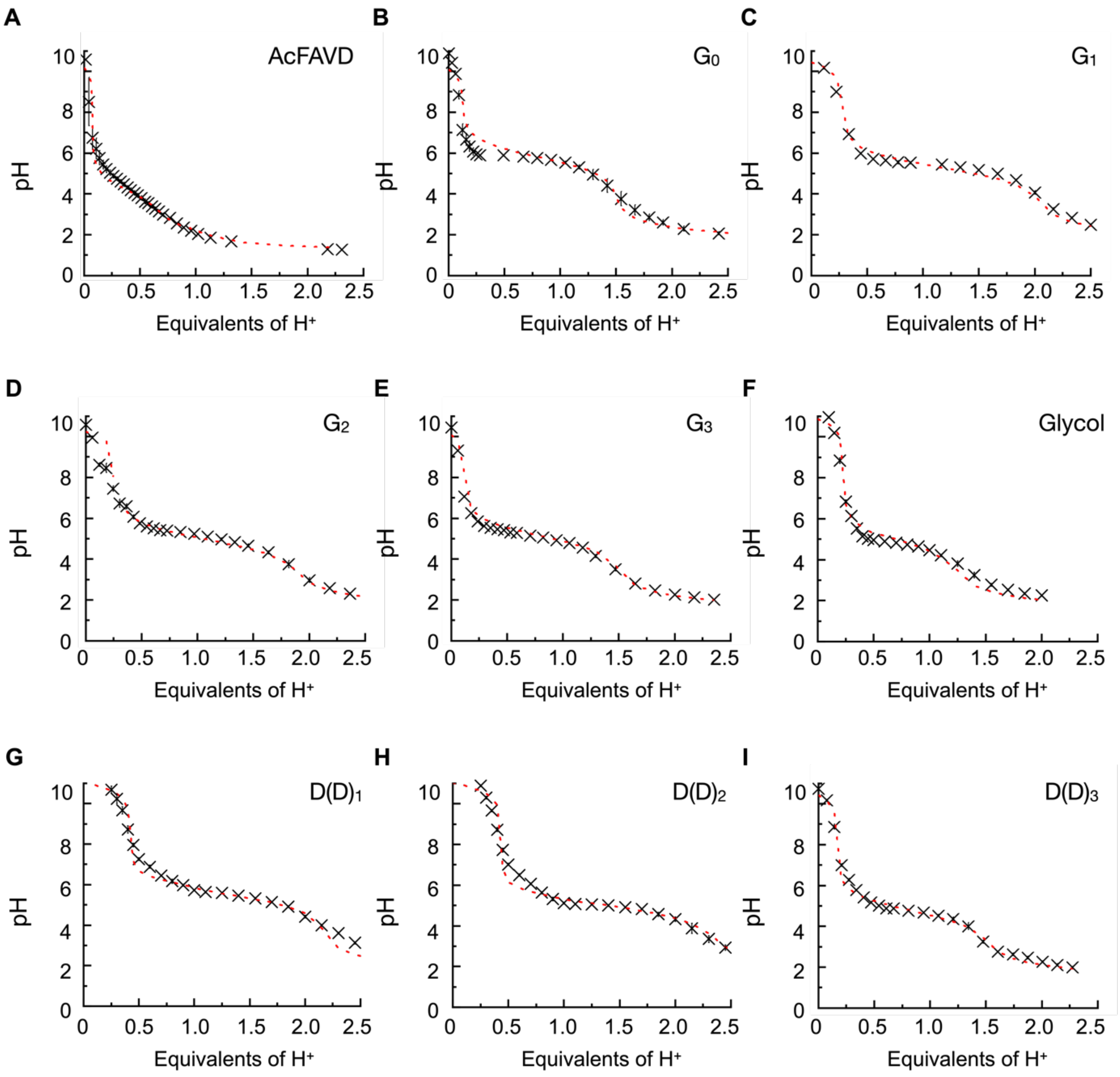

Figure S11: Titration of **A)** 10 mM Ac-FAVD (pK_a α-COOH (Asp): 2.2 ± 0.1, pK_a (COOH of Asp side chain): 4.2 ± 0.1), **B)** 10 mM G_0 (pK_a: 5.8 ± 0.1), **C)** 10 mM G_1 (pK_a: 5.3 ± 0.1), **D)** 10 mM G_2 (pK_a: 5.1 ± 0.1) **E)** 10 mM G_3 (pK_a: 5.1 ± 0.1), **F)** 10 mM Glycol (pK_a: 4.7 ± 0.1), **G)** 10 mM $D(D)_1$ (pK_a: 5.6 ± 0.1), **H)** 10 mM $D(D)_2$ (pK_a: 5.0 ± 0.1), **I)** 10 mM $D(D)_3$ (pK_a: 4.7 ± 0.1) with 0.1 M HCl. Markers represent experimental data. Error bar: n = 3. Dashed lines represent the titration curve fit of the Hyperquad2008 program.[2]

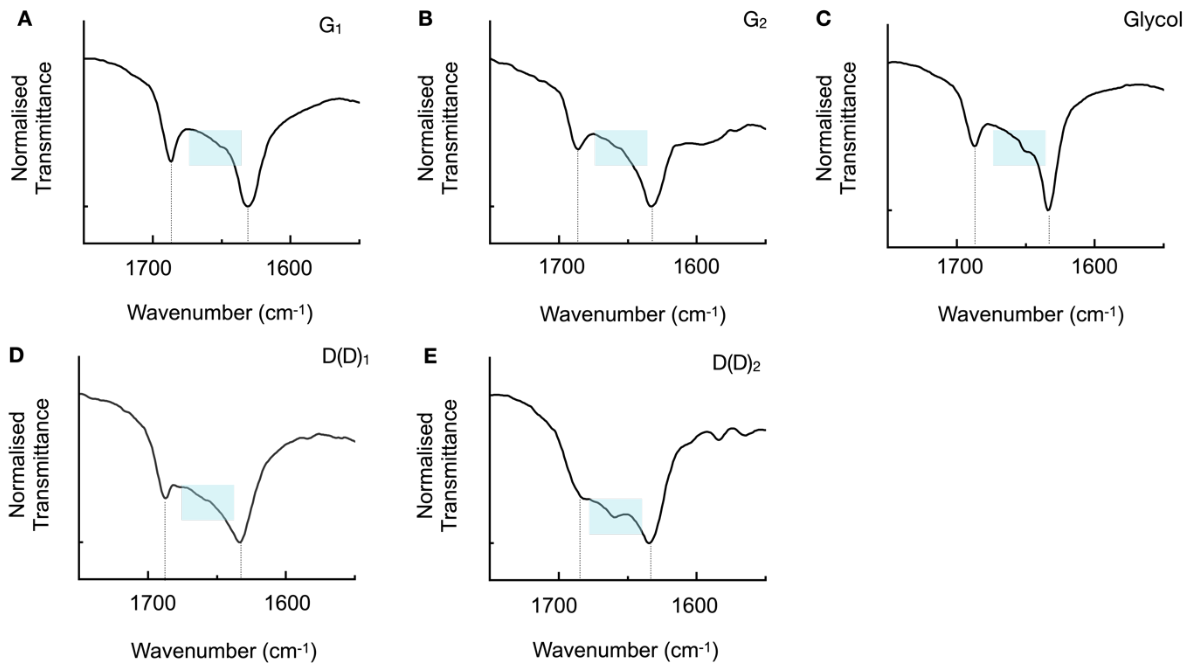

Figure S12: A-D) FT-IR spectra of **A)** G_1, **B)** G_2, **C)** Glycol, **D)** $D(D)_1$, **E)** $D(D)_2$ fueled with EDC at 3 min. Fmoc-OCNHband at 1687/1686/1687/1687/1687 cm^{-1} (dashed line) and amide I band at 1631/1633/1634/1635/1634 cm^{-1} (typical β-sheet, dashed line). The blue box highlights the broad shoulder between 1640-1670 cm^{-1}.

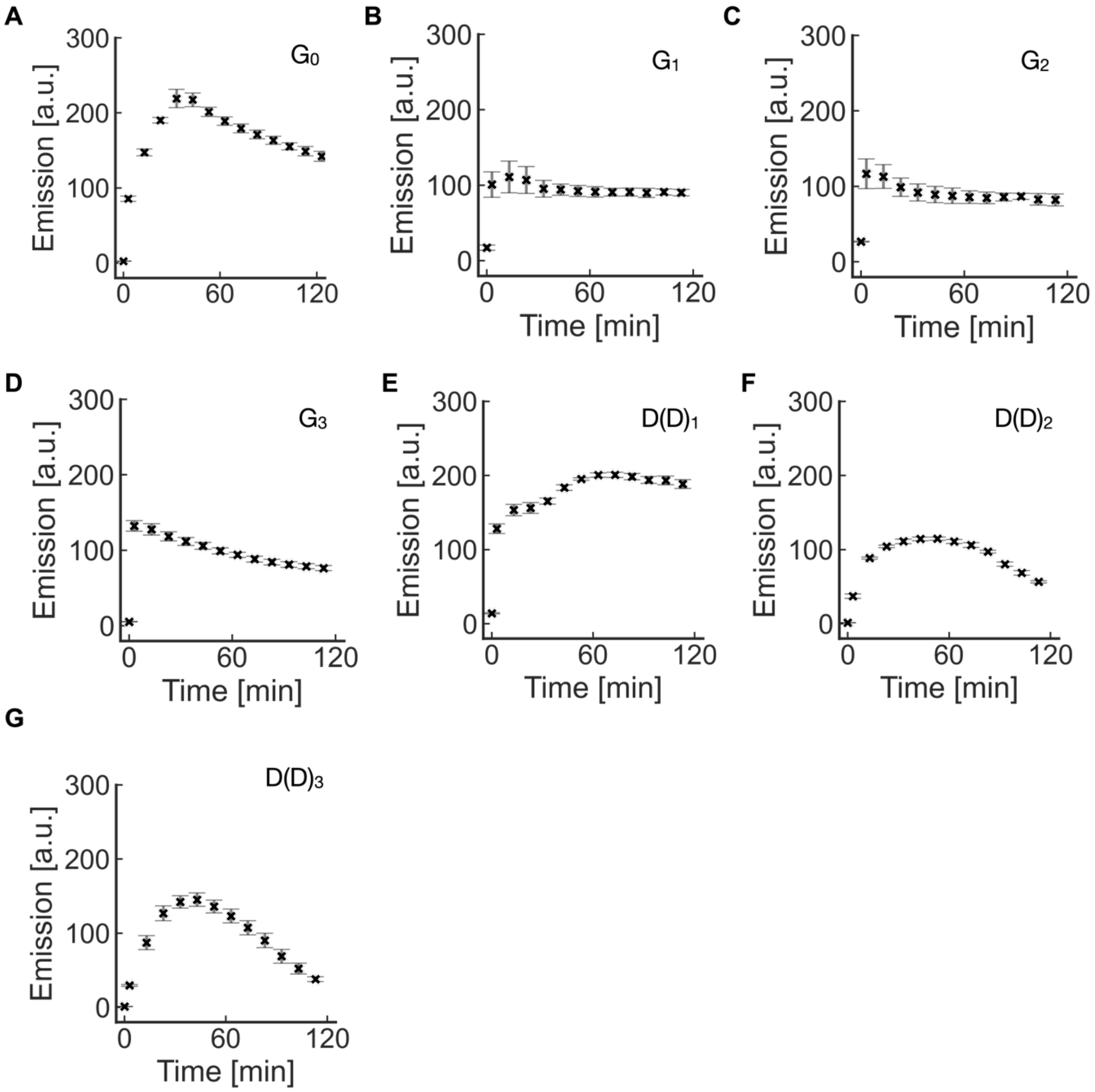

Figure S13: ThT fluorescence intensity at 485 nm after excitation at 440 nm against time as a measure for the presence of β-sheets when **A)** G_0 **B)** G_1 **C)** G_2 **D)** G_3 **E)** $D(D)_1$ **F)** $D(D)_2$ **G)** $D(D)_3$ are fueled with 10 mM EDC, in 200 mM MES buffer, pH 6. Error bar: n = 3.

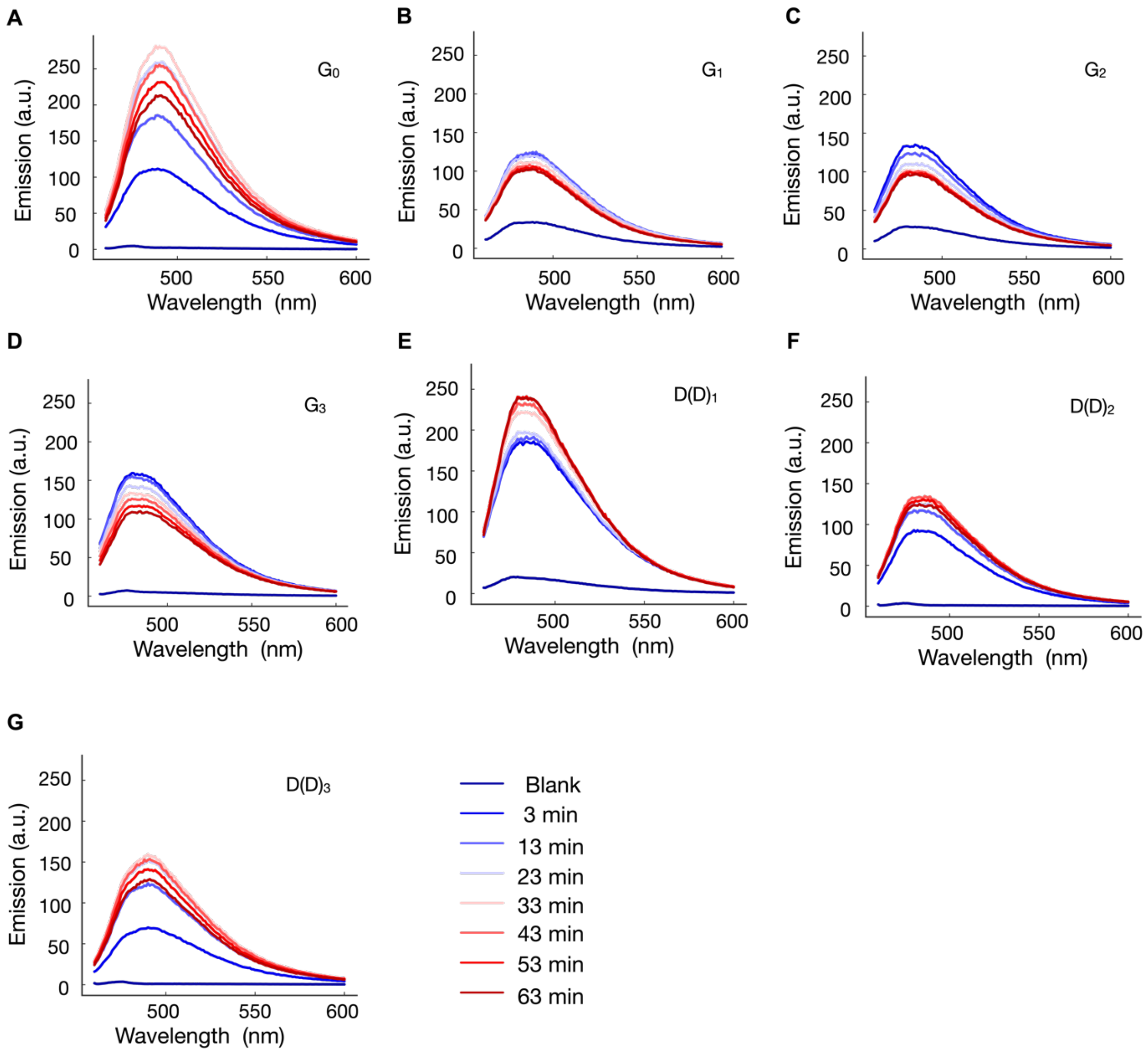

Figure S14: ThT fluorescence spectra over time as a measure for the presence of β-sheets when **A)** G_0 **B)** G_1 **C)** G_2 **D)** G_3 **E)** $D(D)_1$ **F)** $D(D)_2$ **G)** $D(D)_3$ are fueled with 10 mM EDC, in 200 mM MES buffer, pH 6. The spectra are plotted by the mean value of 3 independent experiments.

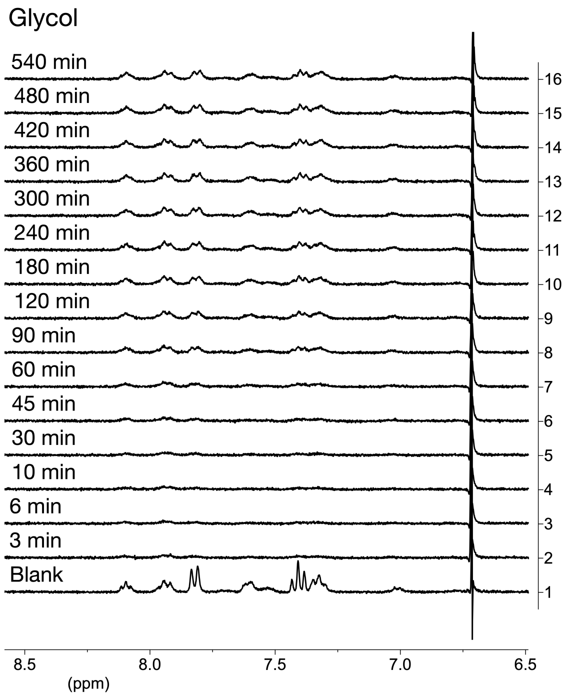

Figure S15: Representative ¹H-NMR spectra`s from 8.50 ppm to 6.50 ppm of 2.5 mM peptide fueled with 50 mM EDC as a function of time of Glycol in 200 mM MES, pH6, 300 MHz. The non-assembled peptide concentration was determined by comparing the integral of the Fmoc-protons at 7.20 – 7.50 ppm to the integral of the internal hydroquinone standard at 6.70 ppm. MestReNova© software.

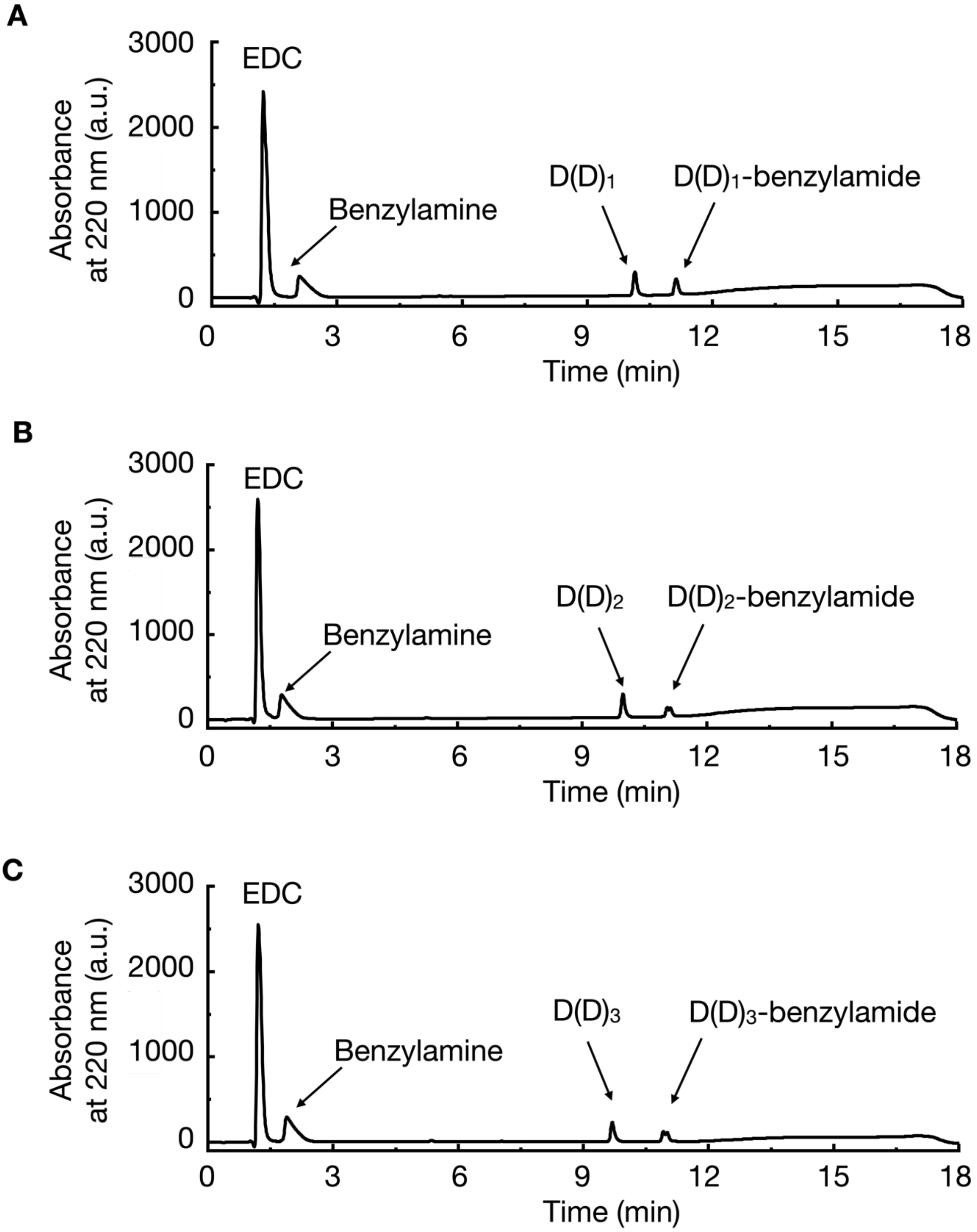

Figure S16: Representative HPLC-chromatogram at 3 min at 220 nm (injection volume: 1 μL) of 2.5 mM **A)** $D(D)_1$, **B)** $D(D)_2$, **C)** $D(D)_3$ quenched 1:1 with 400 mM benzylamine after adding 50 mM EDC.

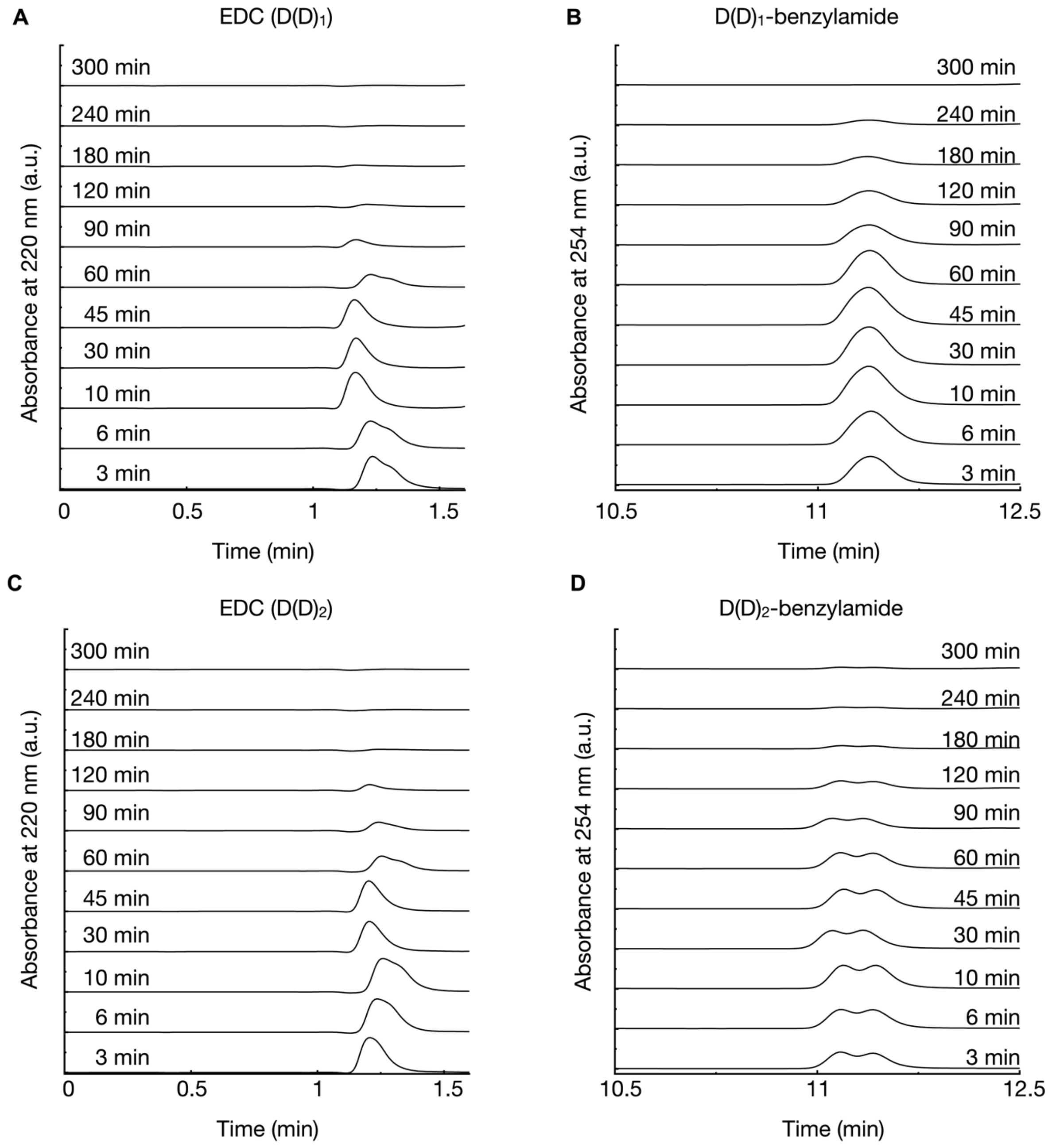

Figure S17: Representative HPLC-chromatograms of EDC-peak over time at 220 nm and benzylamide peak over time at 254 nm (injection volume: 1 µL) of 2.5 mM **A-B)** D(D)$_1$, **C-D)** D(D)$_2$ quenched 1:1 with 400 mM benzylamine after adding 50 mM EDC.

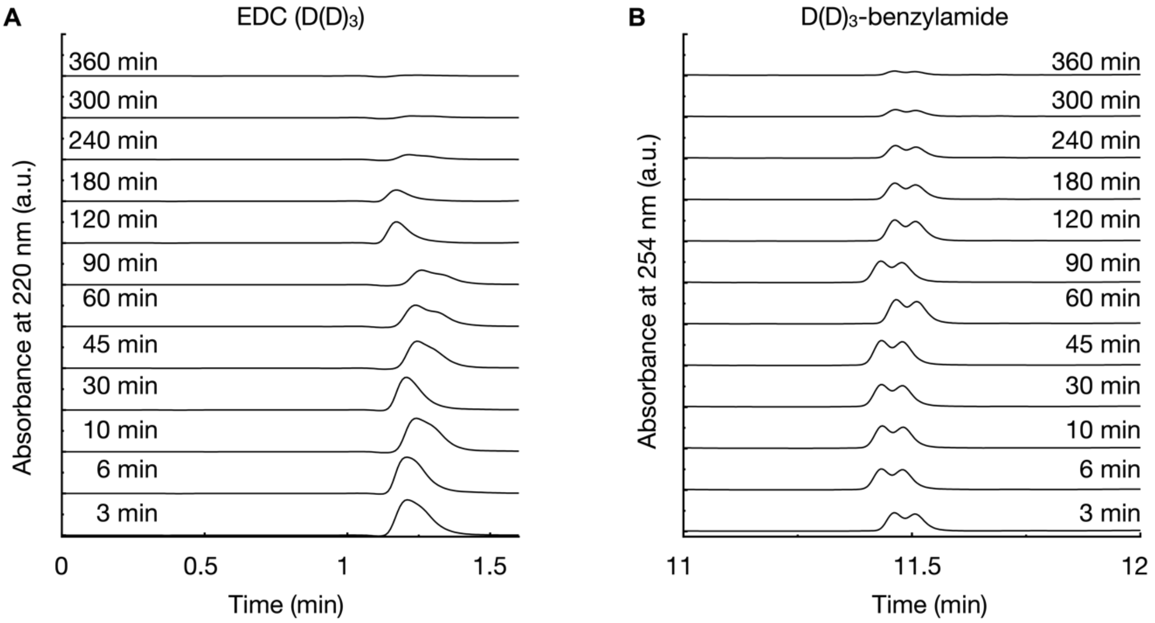

Figure S18: Representative HPLC-chromatograms of EDC-peak over time at 220 nm and benzylamide peak over time at 254 nm (injection volume: 1 µL) of 2.5 mM **A-B)** $D(D)_3$ quenched 1:1 with 400 mM benzylamine after adding 50 mM EDC.

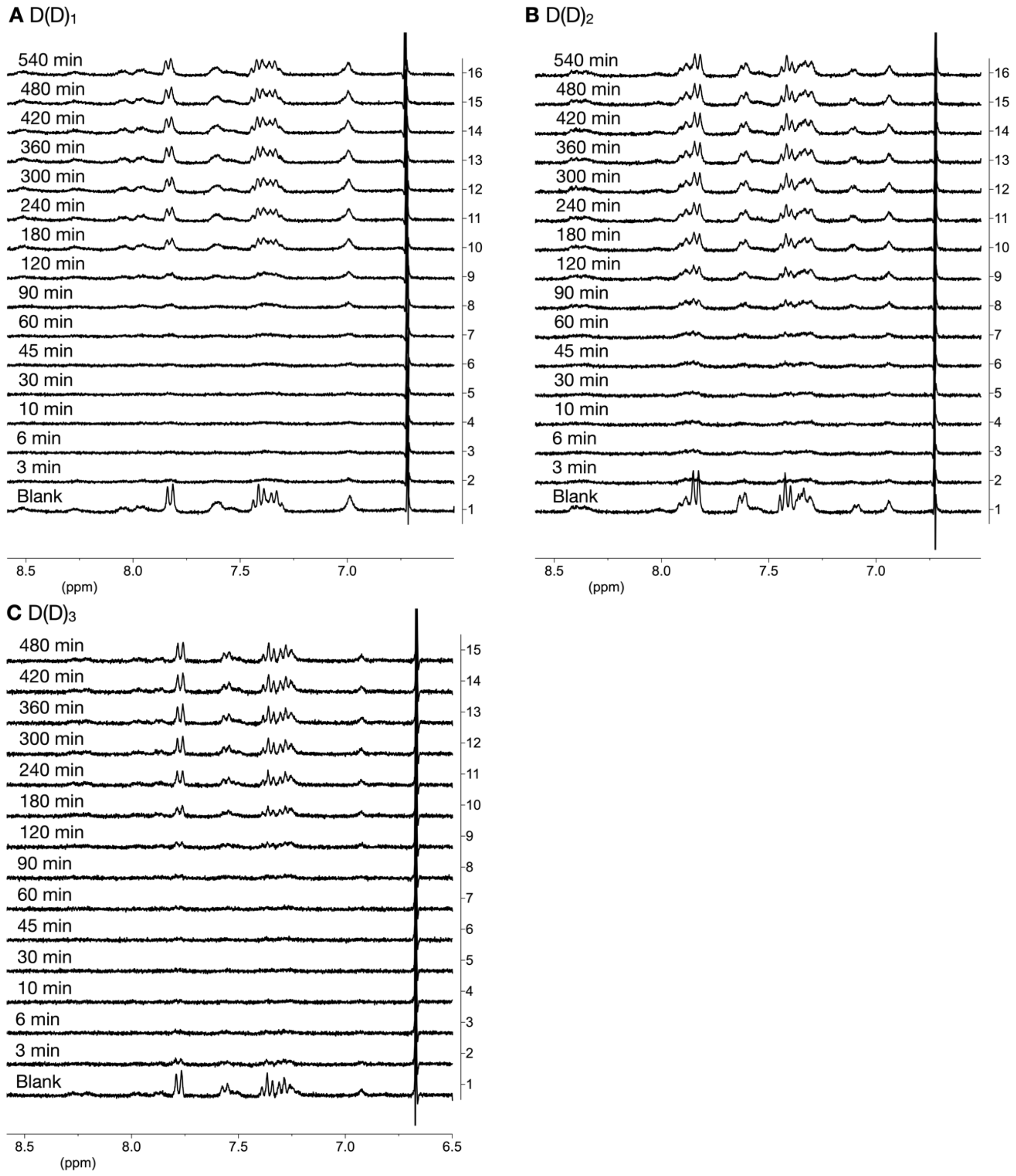

Figure S19: Representative ^{1}H-NMR spectra`s from 8.50 ppm to 6.50 ppm of 2.5 mM peptide fueled with 50 mM EDC as a function of time of **A)** $D(D)_1$ **B)** $D(D)_2$ **C)** $D(D)_3$ in 200 mM MES, pH6, 300 MHz. The non-assembled peptide concentration was determined by comparing the integral of the Fmoc-protons at 7.20 – 7.50 ppm to the integral of the internal hydroquinone standard at 6.70 ppm. MestReNova© software.

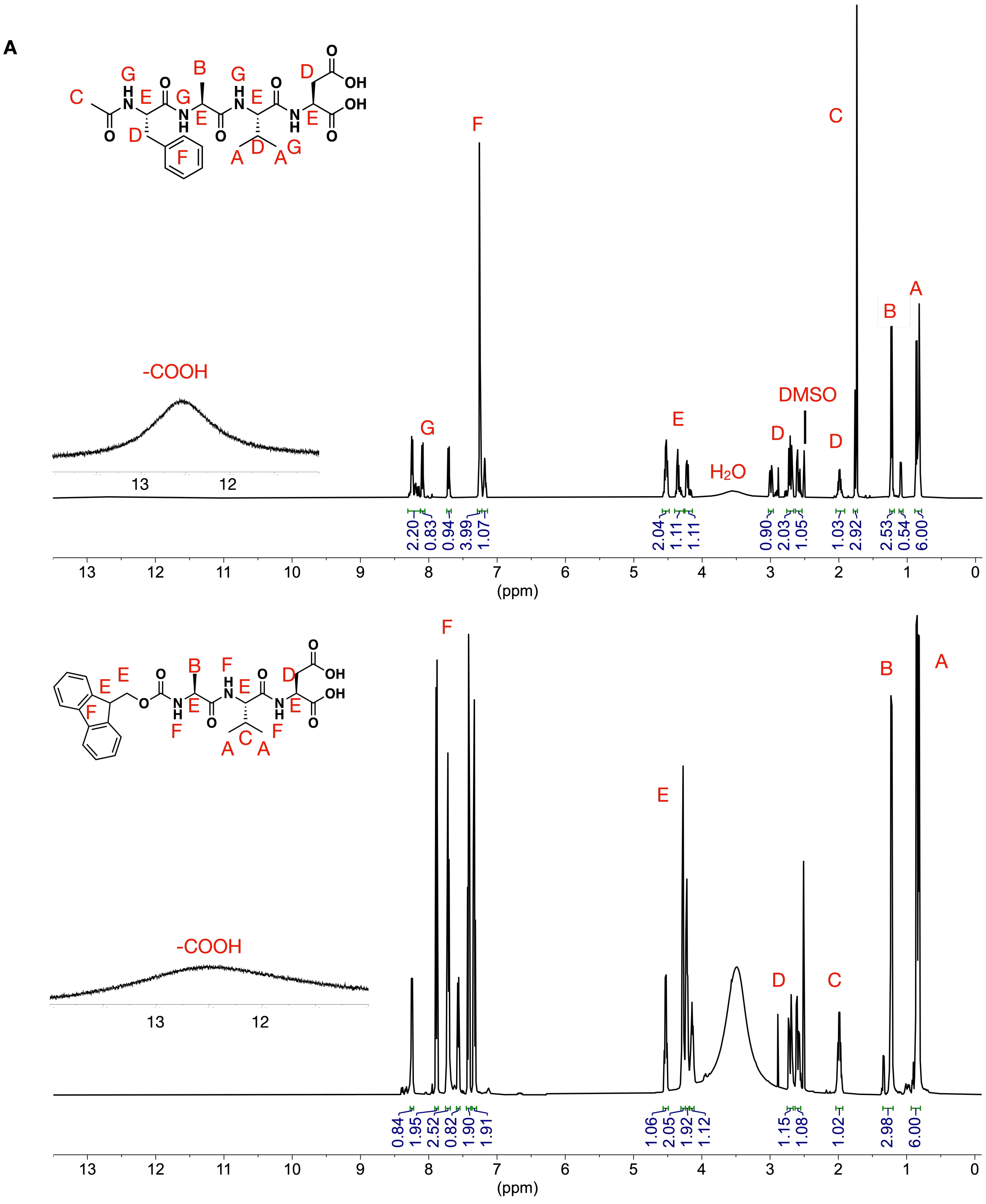

Figure S20: ^{1}H-NMR spectrum of **A)** Ac-FAVD and **B)** G_0 in DMSO-d_6, 500 MHz.

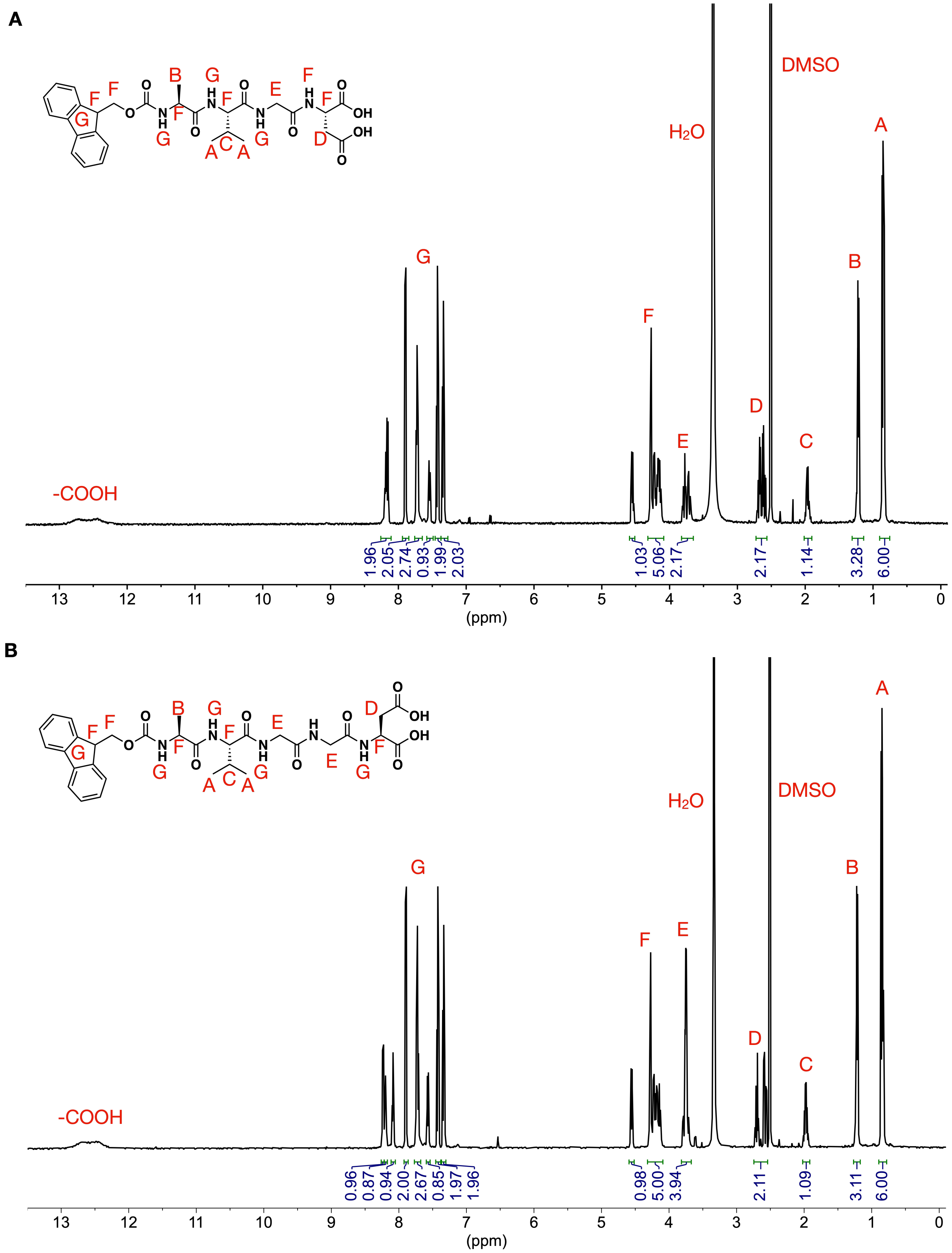

Figure S21: ^{1}H-NMR spectrum of **A)** G_1 and **B)** G_2 in DMSO-d_6, 500 MHz.

A

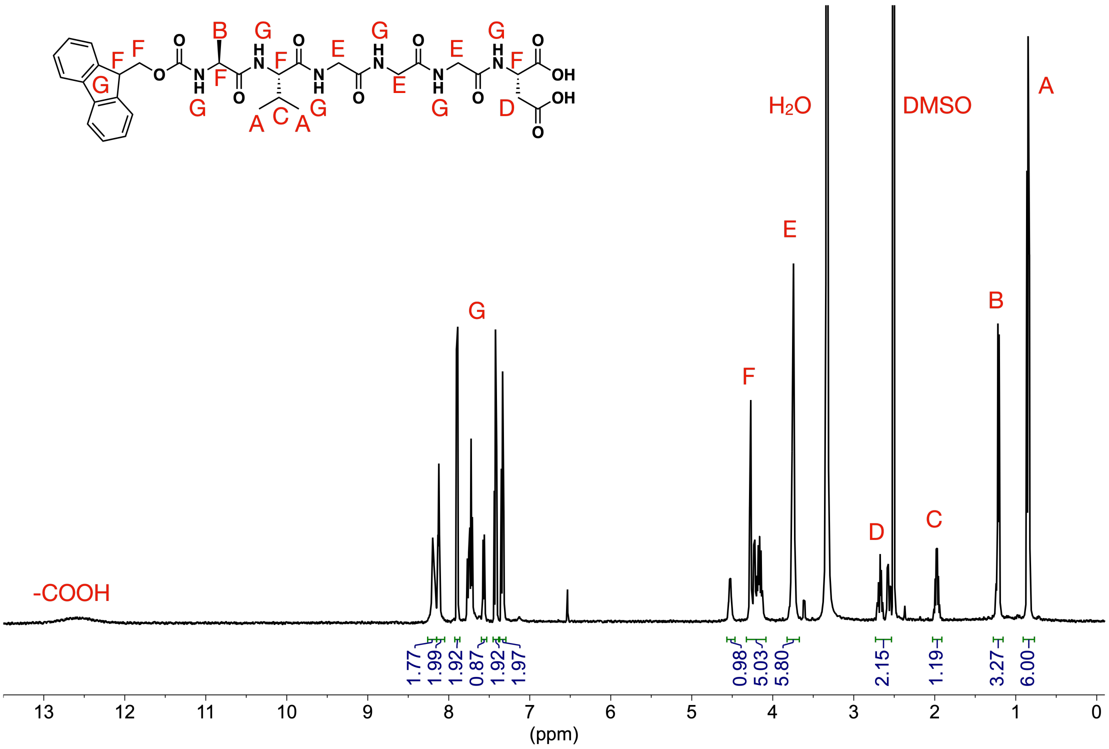

B

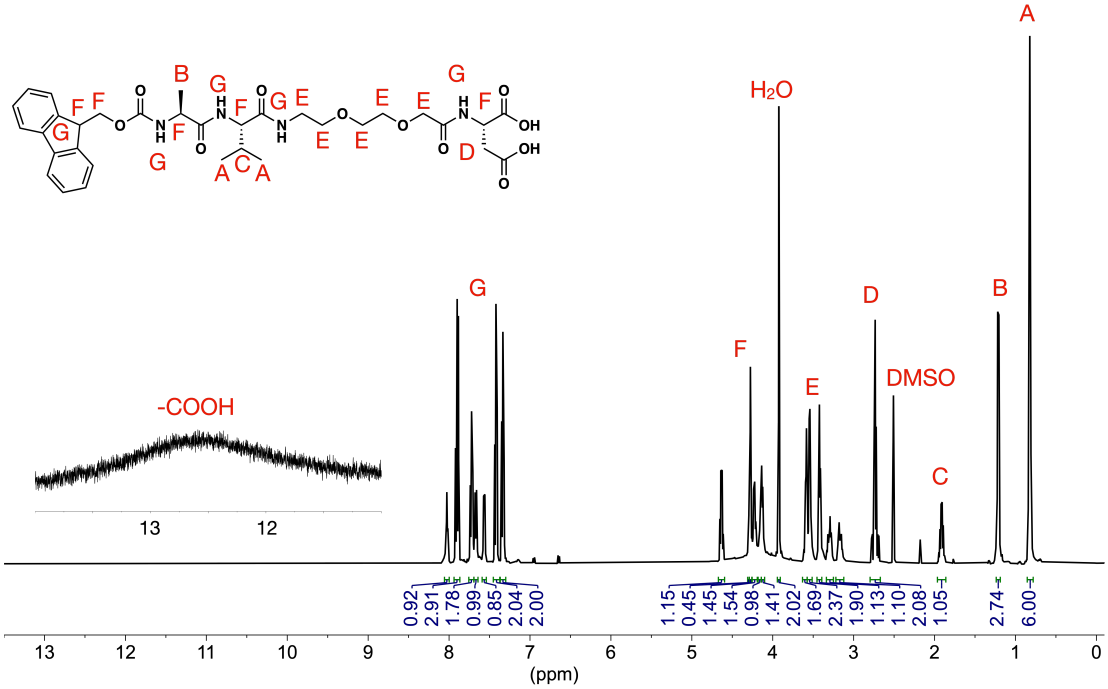

Figure S22: ^{1}H-NMR spectrum of **A)** G_3 and **B)** Glycol in DMSO-d_6, 500 MHz.

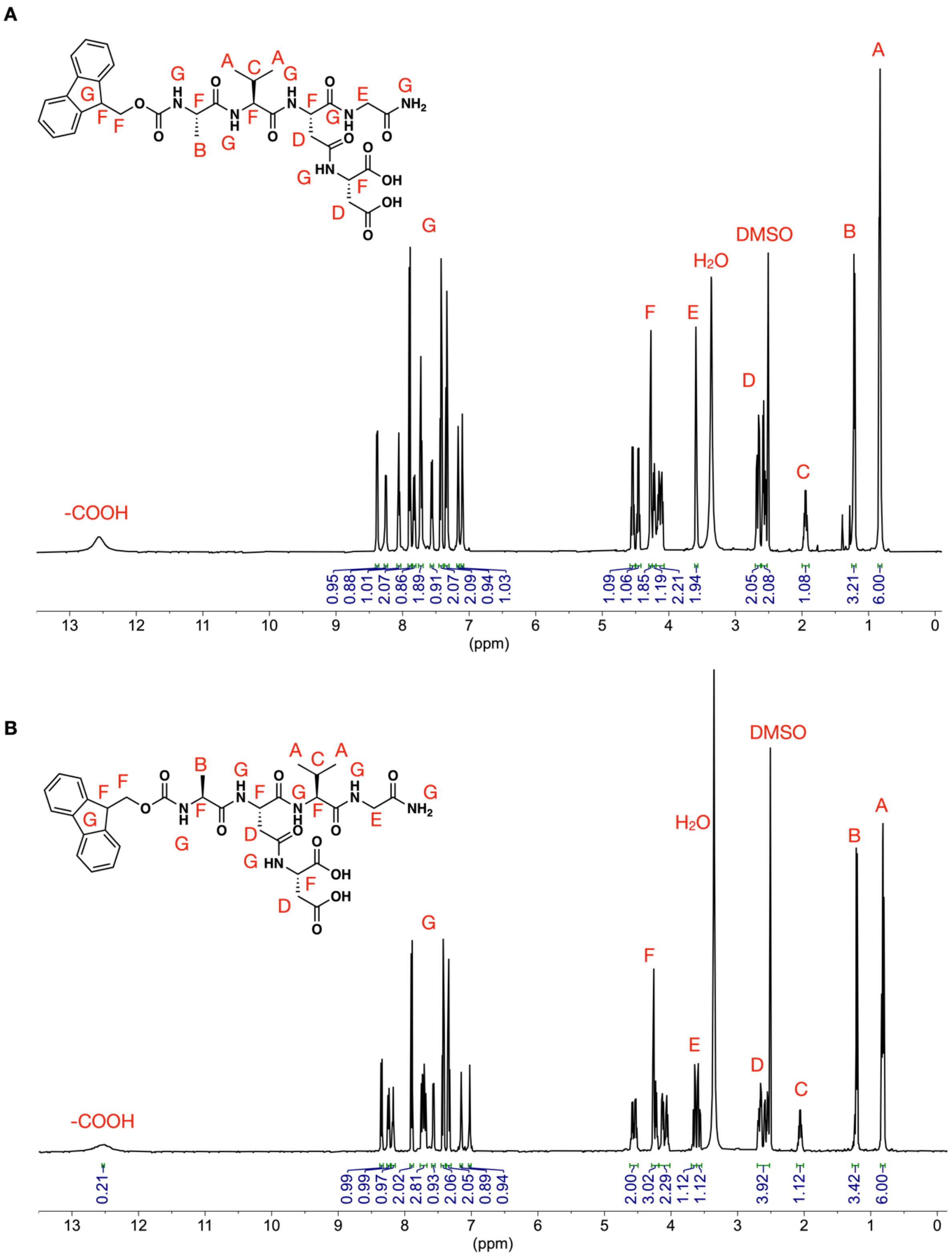

Figure S23: ^{1}H-NMR spectrum of **A)** D(D)$_1$ and **B)** D(D)$_2$ in DMSO-d$_6$, 500 MHz.

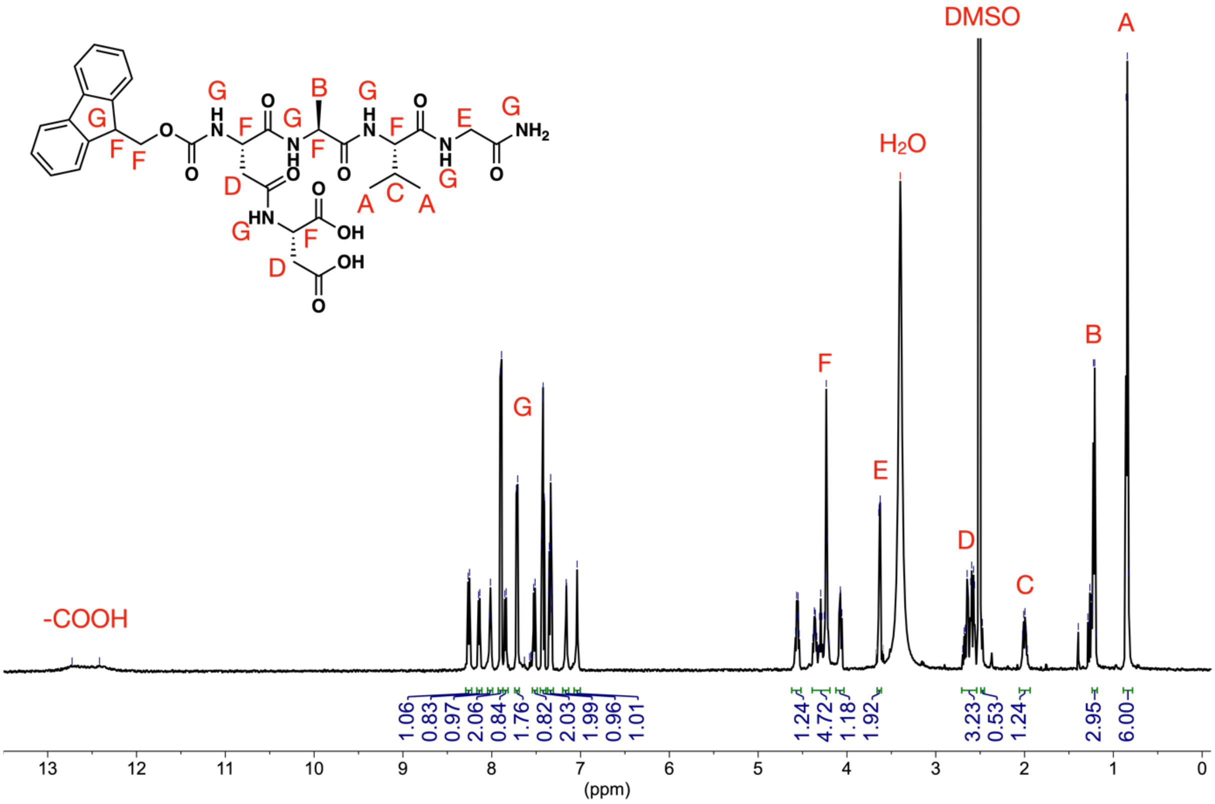

Figure S24: ^{1}H-NMR spectrum of D(D)$_3$ in DMSO-d$_6$, 500 MHz.

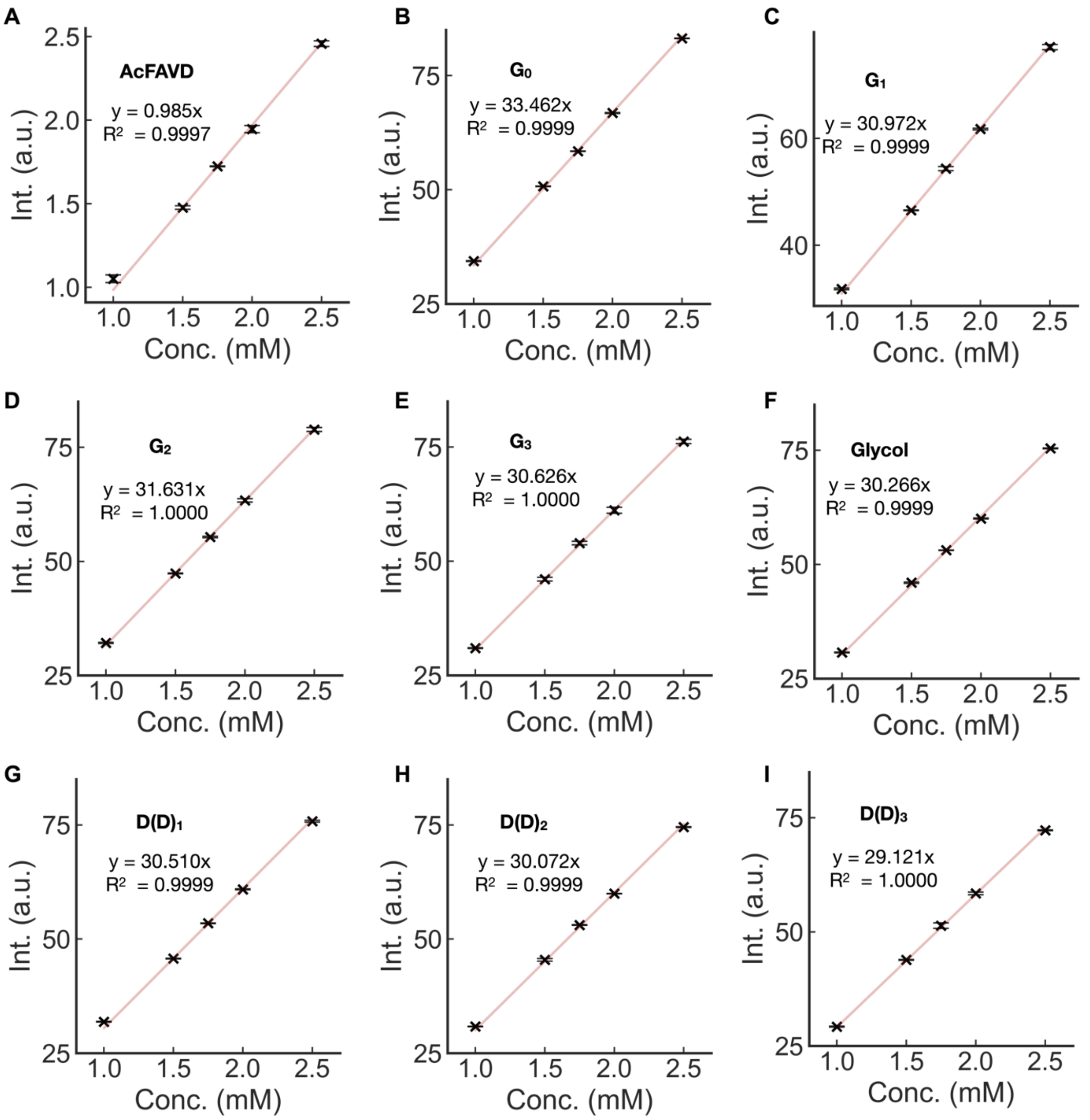

Figure S25: Calibration curves of 1 µL injection volume at 254 nm **A)** Ac-FAVD (2 µL injection volume, 220 nm), **B)** G_0 **C)** G_1 **D)** G_2 **E)** G_3 **F)** Glycol **G)** $D(D)_1$ **H)** $D(D)_2$ **I)** $D(D)_3$. Error bar: n = 3.

References

1. F. Schnitter and J. Boekhoven, A Method to Quench Carbodiimide-Fueled Self-Assembly, *ChemSystemsChem*, 2021, **3**, e2000037.
2. P. Gans, A. Sabatini and A. Vacca, Investigation of equilibria in solution. Determination of equilibrium constants with the HYPERQUAD suite of programs, *Talanta*, 1996, **43**, 1739-1753.
3. X. Chen, M. Stasi, J. Rodon-Fores, P. F. Grossmann, A. M. Bergmann, K. Dai, M. Tena-Solsona, B. Rieger and J. Boekhoven, A Carbodiimide-Fueled Reaction Cycle That Forms Transient 5(4H)-Oxazolones, *J Am Chem Soc*, 2023, **145**, 6880-6887.
4. X. Chen, H. Soria-Carrera, O. Zozulia and J. Boekhoven, Suppressing catalyst poisoning in the carbodiimide-fueled reaction cycle, *ChemRxiv*, 2023, DOI: 10.26434/chemrxiv-2023-v8v53.
5. L. S. Kariyawasam and C. S. Hartley, Dissipative Assembly of Aqueous Carboxylic Acid Anhydrides Fueled by Carbodiimides, *J Am Chem Soc*, 2017, **139**, 11949-11955.
6. M. Tena-Solsona, B. Rieß, R. K. Grötsch, F. C. Löhrer, C. Wanzke, B. Käsdorf, A. R. Bausch, P. Müller-Buschbaum, O. Lieleg and J. Boekhoven, Non-equilibrium dissipative supramolecular materials with a tunable lifetime, *Nature Communications*, 2017, **8**, 15895.
